# The role of SNP-loop diuretic interactions in hypertension across ethnic groups in HyperGEN

**DOI:** 10.3389/fgene.2013.00304

**Published:** 2013-12-25

**Authors:** Lisa de Las Fuentes, Yun Ju Sung, Karen L. Schwander, Sonia Kalathiveetil, Steven C. Hunt, Donna K. Arnett, D. C. Rao

**Affiliations:** ^1^Cardiovascular Division, Department of Medicine, Washington University School of MedicineSt. Louis, MO, USA; ^2^Division of Biostatistics, Washington University School of MedicineSt. Louis, MO, USA; ^3^Division of Cardiovascular Genetics, Department of Internal Medicine, University of Utah School of MedicineSalt Lake City, UT, USA; ^4^Department of Epidemiology, University of Alabama at BirminghamBirmingham, AL, USA

**Keywords:** blood pressure, hypertension, loop diuretic, gene-drug interaction, genome-wide association, african americans, european americans

## Abstract

Blood pressure (BP) is significantly influenced by genetic factors; however, less than 3% of the BP variance has been accounted for by variants identified from genome-wide association studies (GWAS) of primarily European-descent cohorts. Other genetic influences, including gene-environment (GxE) interactions, may explain more of the unexplained variance in BP. African Americans (AA) have a higher prevalence and earlier age of onset of hypertension (HTN) as compared with European Americans (EA); responses to anti-hypertensive drugs vary across race groups. To examine potential interactions between the use of loop diuretics and HTN traits, we analyzed systolic (SBP) and diastolic (DBP) blood BP from 1222 AA and 1231 EA participants in the Hypertension Genetic Epidemiology Network (HyperGEN). Population-specific score tests were used to test associations of SBP and DBP, using a panel of genotyped and imputed single nucleotide polymorphisms (SNPs) for AA (2.9 million SNPs) and EA (2.3 million SNPs). Several promising loci were identified through gene-loop diuretic interactions, although no SNP reached genome-wide significance after adjustment for genomic inflation. In AA, SNPs in or near the genes *NUDT12*, *CHL1, GRIA1*, *CACNB2*, and *PYHIN1* were identified for SBP, and SNPs near *ID3* were identified for DBP. For EA, promising SNPs for SBP were identified in *ESR1* and for DBP in *SPATS2L* and *EYA2.* Among these SNPs, none were common across phenotypes or population groups. Biologic plausibility exists for many of the identified genes, suggesting that these are candidate genes for regulation of BP and/or anti-hypertensive drug response. The lack of genome-wide significance is understandable in this small study employing gene-drug interactions. These findings provide a set of prioritized SNPs/candidate genes for future studies in HTN. Studies in more diversified population samples may help identify previously missed variants.

## Introduction

High blood pressure (BP) is the leading cause of death in the United States, contributing to a wide spectrum of cardiovascular, cerebrovascular, and renal diseases (Chobanian et al., 2003). In the United States, one in three adults has high BP, with a mortality rate of 18.3 per 100,000 individuals annually. Public health costs due to hypertension (HTN) and its sequelae are approximately $51 billion per year (Roger et al., 2012). Among US adults, the age-adjusted prevalence of HTN is 42.5% in African Americans (AA), 26.1% for Mexican Americans, and 29.1% for Non-Hispanic European Americans (EA). This disparity is greater for HTN than for other cardiovascular risk factors including dyslipidemia and diabetes (Fryar et al., 2010). In past years, genetic studies of HTN and BP were largely limited to cohorts of European ancestry. However, it is still unknown the extent to which findings discovered in a European-based population are applicable to other race/ethnic groups (Rosenberg et al., 2010).

Although a moderate portion of the between-individual variance of HTN-related traits are explained by genetic factors, identifying the loci responsible for BP regulation has remained frustrating. The heritability of BP ranges from about 30–45% in pedigree data to as high as 60% in twin studies (Biron et al., 1976; Feinleib et al., 1977; Rotimi et al., 1999; Snieder et al., 2003; Hottenga et al., 2005; Mitchell et al., 2005). Heritability estimates vary depending on the specific trait (for example, systolic BP, diastolic BP, and HTN) and the means by which these traits are ascertained. Heritability estimates derived from non-European cohorts are similar to or higher than in European cohorts (Rotimi et al., 1999; Bochud et al., 2005; Zhang et al., 2009). Yet despite the substantial estimated heritability and sample size in excess of 200,000 subjects, common variants identified by genome-wide association (GWA) studies explain less than 3% of inter-individual BP variation (International Consortium for BP GWAS et al., 2011). While the reasons for this “missing heritability” have been explored in detail elsewhere (Manolio et al., 2009; Zhang et al., 2010), it is important to consider that the majority of GWA studies ascertain traits on the background of ongoing drug therapy. Therefore, in lieu of statistical models that consider only the genetic main effects, analyses incorporating gene-drug interactions may increase the yield for findings of association.

According to guidelines recommended by the Joint National Committee on Prevention, Detection, Evaluation, and Treatment of High BP, thiazide and thiazide-like diuretics should be considered first-line antihypertensive drug therapy for patients with uncomplicated HTN in the absence of other “compelling” comorbid indications such as diabetes and heart failure (Chobanian et al., 2003). This recommendation was based largely on the results of the Antihypertensive and Lipid-Lowering Treatment to Prevent Heart Attack Trial (ALLHAT) in which use of a diuretic was associated with reduced incidence of heart failure compared to the angiotensin-converting enzyme inhibitor and the calcium-channel blocker (ALLHAT Collaborative Research Group, 2002). However, in addition to the disparity in the prevalence of HTN, differences in drug response have also been noted. For example, African-American race is associated with significantly greater systolic BP (SBP) and/or diastolic BP (DBP) responses to thiazide and loop diuretics compared to European-American patients in small studies (Chapman et al., 2002; Wu et al., 2005). Population-based differences may be attributable, at least in part, to differences in the genetic architecture of BP between world populations. Therefore, current efforts attempting to increase representation from other continental populations may improve the generalizability of genetic studies to diverse patient populations.

Loop diuretics such as furosemide and bumetanide lower BP by blocking the action of the *SLC12A1* (NKCC2) sodium-potassium-chloride channel in kidney, resulting in decreased reabsorption of sodium, potassium, and chloride, and hence water, from the urine. Bartter's Syndrome Type 1 was identified by linkage analysis to be the result of homozygous or compound heterozygous non-functional mutations in the *SLC12A1* gene; this rare disorder is characterized by neonatal hypotension, volume depletion, and increased risk for death (Simon et al., 1996). Loop diuretics also inhibit the *SLC12A5* (NKCC1), a homolog that is more widely expressed in the brain (Kanaka et al., 2001). The potential for genetic variants to modulate drug response extends beyond the actual molecular targets of the drug and may include variants in proteins controlling intestinal absorption, enzymatic biotransformation, binding to carrier proteins, and renal elimination (Vormfelde et al., 2003).

Here we consider whether SNP-loop diuretic interactions play a role in the genetic architecture of BP in African- and European-American families in the HyperGEN study. The identification of genetic variants that affect drug response could help practitioners to individualize medical therapy with the goal of optimizing benefit and reducing adverse effects both within and across populations.

## Materials and methods

### Study sample

HyperGEN is a multicenter family-based study to research the genetic causes of HTN and related conditions (Williams et al., 2000). Hypertensive sibships were enrolled from four separate field centers from 1995 to 2005: Minneapolis, MN; Salt Lake City, UT; Forsyth County, NC; and Birmingham, AL. The available dataset was derived from 1258 subjects in 467 African-American families and 1270 subjects in 299 European-American families. Subjects with missing covariates (age, sex, and body mass index [BMI]) and/or drug exposure (loop diuretics) were excluded. Individuals with HTN onset past age 60 or secondary to primary kidney disease were also excluded (Williams et al., 2000). Given loop diuretics are frequently prescribed to control symptoms in patients with reduced left ventricular systolic function, two analysis models were considered. For the primary analysis, we used the “Continuous-Covariate” model which included left ventricular ejection fraction (LVEF) as a continuous covariate; 1222 subjects in 459 African-American families and 1231 subjects in 299 European-American families were available. To test for a possible confounding association between the uses of loop diuretics in subjects with low LVEF, the secondary “All Subjects” model included all subjects regardless of the availability or value of LVEF; 1249 subjects in 466 African-American families and 1267 subjects in 299 European-American families were available. Supine BPs and LVEF were measured as previously described (Williams et al., 2000; Kizer et al., 2004). The institutional review board at each field center gave approval for the study protocol and informed consent procedure.

### Genotyped and imputed data

Most of the African-American subjects (*N* = 1083) were genotyped using the Affymetrix Genome-wide Human SNP array 6.0 [909,622 SNPs; Birdseed Genotype Calling Algorithm (Korn et al., 2008)]. The remaining African-American (*N* = 175) and all 1270 European-American subjects were genotyped using the Affymetrix Genome-wide Human SNP array 5.0 (443,816 SNPs; Bayesian Robust Linear Model with Mahalanobis distance classifier [BRLMM] calling algorithm). Genomic data was quality controlled separately by race and by array as follows: samples were removed for quality-related problems; SNPs were removed if monomorphic, identified as an Affymetrix “house-keeping” SNP, missing chromosomal location, or located on a mitochondrial or sex chromosome; PLINK (Purcell et al., 2007) was used to find and correct all non-Mendelian inheritance errors (performed in a dataset integrating both arrays for the AA datasets); and SNPs with missing rates >5%, minor allele frequency <1%, or Hardy Weinberg *p*-value <10^−6^ were excluded. An account of genotyped SNPs remaining following quality control is shown in Supplement Table 1.

Genotype imputation was performed separately within each race by the HyperGEN Data Coordinating Center using the post-quality controlled genotype data for HyperGEN African-American (846,813 genotyped SNPs) and European-American (358,327 genotyped SNPs) subjects. Data from unrelated African-American individuals (200 with the lowest GWAS missing rate) were chosen from a total of 467 African-American HyperGEN families to estimate the imputation model parameters. Using MACH software and the estimated imputation model parameters, ~3.01 million HapMap SNPs (release 22, phasing data, “revised union” of CEU and YRI populations) were imputed in the African-American cohort. For association analysis, we used a dataset of 2,943,641 genotyped and imputed SNPs, after excluding SNPs that had imputation quality measure (*R*^2^) < 0.3, minor allele frequency <1%, or Hardy Weinberg *p*-value <10^−6^. Genotypes were similarly imputed in the European-American cohort using ~2.54 million HapMap SNPs (release 22, phasing data, CEU population). We used a dataset of 2,367,693 genotyped and imputed SNPs using the same exclusion criteria. Association analyses were performed separately using genotyped and imputed SNPs and then results combined; when both genotyped and imputed data was available at any locus, the genotyped data was retained preferentially. Genomic information was obtained from the National Center for Biotechnology Information human genome assembly (March 2006, build 36.1, hg18). A locus was defined as a cluster of SNPs within 100kb of the SNP with the lowest *P*-value in the region.

### Analysis approach

Population characteristics were expressed as percentages or mean ± standard deviation. The EIGENSOFT package (Patterson et al., 2006) was used to estimate a set of 10 principal components (PCs) for the AA subjects from 382,671 SNPs (representing the intersection of the Affymetrix Genome-Wide Human SNP 5.0 and 6.0 Array SNPs). The PCs were estimated from unrelated subjects chosen from each family; the 10 PC's were then predicted for the remaining subjects. Since HyperGEN is a family study, a linear mixed model with a random polygenic component was used to account for phenotypic correlations among relatives. We used GenABEL (Aulchenko et al., 2007) and ProbABEL (Aulchenko et al., 2010) packages, components of the ABEL suite, and performed a two-step score test approximation, following the developer's recommendation for the analysis of family data. In the first step, GenABEL was used to run a polygenic model, which provides maximum likelihood estimates (MLEs) of our covariate effects (e.g., age, age^2^, age^3^, sex, BMI, and LVEF), residual variance, and variance-covariance matrix for the SBP (or DBP) phenotype. Analyses of the African-American cohort also included 10 principal components of the GWAS SNPs representing population substructure as a co-variate to control type I error rates due to population stratification (Shriner et al., 2011). In the second step, the score tests for SNP and medication main effects (assuming an additive model), as well as their interaction effects, were performed in ProbABEL using the MLE of the other parameters created from the first step.

MERLIN also internally performs association analysis using this two-step score approach and provides identical results for the main effect, but it is more straightforward to use the ABEL suite for interaction analysis. Because both the ABEL suite and MERLIN take account for phenotypic correlation across family members using the kinship matrix, they are more sophisticated than a mixed effects model with random effect for family relatedness. For example, in a mixed model the phenotypic correlation of half siblings is the same as the correlation of full siblings, whereas in the ABEL suite the correlation of half siblings is half the correlation of full siblings. The effect of loop diuretics was coded as dummy variable (0/1), whose main effect was included along with SNP-drug interaction effect in the association analysis for SBP and DBP. For each SNP, the interaction effect coefficients were tested in the presence of the SNP main effect and medication main effect. The exposure group consisted of subjects taking loop diuretics, either alone or in combination with other antihypertensive drugs. The comparison group consisted of subjects not taking loop diuretics, although other antihypertensive drugs were permitted. A Wald test was computed to determine the significance using chi-squared statistics with one degree of freedom. The “Continuous-Covariate” model included LVEF as a continuous covariate. Similarly, the “All Subjects” model was performed using the entire cohort without consideration of the availability or value of LVEF.

Quantile-quantile (QQ) plots were performed for each race/trait combination to assess deviation from the normal distribution; the genomic control inflation (lambda) factor was calculated. Manhattan plots were generated in SAS (Version 9.3, SAS Institute, Cary, North Carolina, USA), with the y-axis indicating −log_10_(*P*) values and the x-axis plots the physical position of the SNPs in genomic order, by chromosome and chromosome position. Plots were created for the SNP main effect in the presence of the medication main effect and interaction effect, as well as for the interaction effect in the presence of the SNP and medication main effects. Regional association plots were generated to highlight chromosomal regions with a clustering of SNPs with suggestive association using LocusZoom software, available at http://csg.sph.umich.edu/locuszoom/. The linkage disequilibrium (LD) (r^2^) was calculated from the hg18/HapMap Phase II YRI population for AA and hg18/HapMap Phase II CEU population for EA. SNPs (circles) are colored according to their level of LD of each SNP with the index SNP (purple diamond) selected as the most significant SNP in the region. Gray points are SNPs without available LD information.

## Results

Compared to the European-American cohort, the African-American cohort tended to be older and more predominantly female, with higher BMI, SBP, and DBP. Loop diuretics were used by 96 (7.9%) African-American and by 48 (3.9%) EA subjects (Table 1). LVEF was missing from 27 AA and 36 EA; these individuals were excluded from the “Continuous-Covariate” models but included in the “All Subjects” models. The prevalence of low EF (LVEF ≤45%) was low (3.8% in AA, and 3.2% in EA).

**Table 1:** Descriptive statistics of the study population.^*^

**Characteristics**	**African- American Cohort**	**European- American Cohort**
Sample size, *n*	1222	1231
Male, *n* (%)	398 (33%)	613 (50%)
Loop diuretic use, *n* (%)	96 (7.9 %)	48 (3.9%)
Age, years	45 ± 13	50 ± 14
Body mass index, kg/m^2^	32.4 ± 7.9	29.3 ± 6.0
Systolic blood pressure, mm Hg	129 ± 22	123 ± 19
Diastolic blood pressure, mm Hg	74 ± 12	71 ± 10
Left ventricular ejection fraction, %	61 ± 8	62 ± 8
% EF ≥ 45	1175 (96.2%)	1192 (96.8%)

* For “Continuous-Covariate” model. % EF ≥ 45, percent with left ventricular ejection fraction ≥45%.

**Figure 1:**
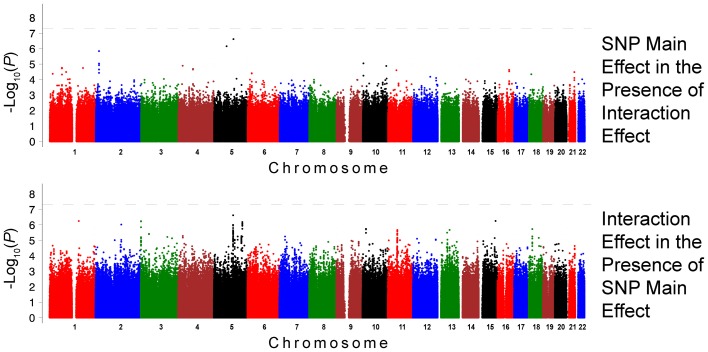
**Association Results for SBP for African Americans: Main and Interaction Effects with Loop Diuretics**.

**Figure 2:**
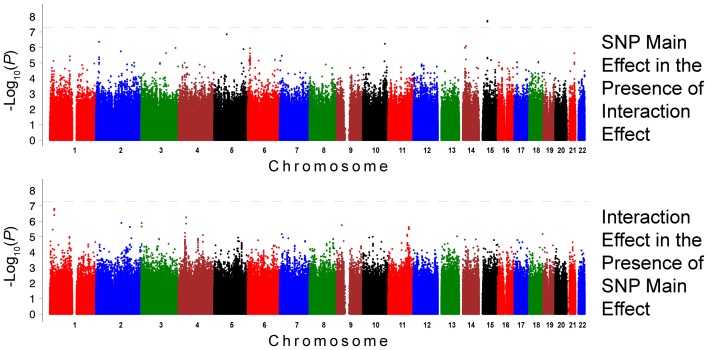
**Association Results for DBP for African Americans: Main and Interaction Effects with Loop Diuretics**.

**Figure 3:**
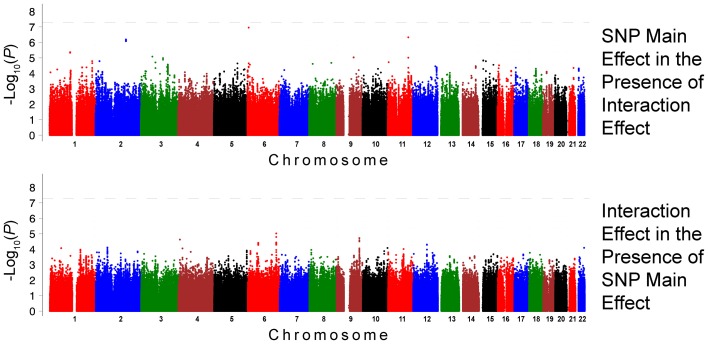
**Association Results for SBP for European Americans: Main and Interaction Effects with Loop Diuretics**.

**Figure 4:**
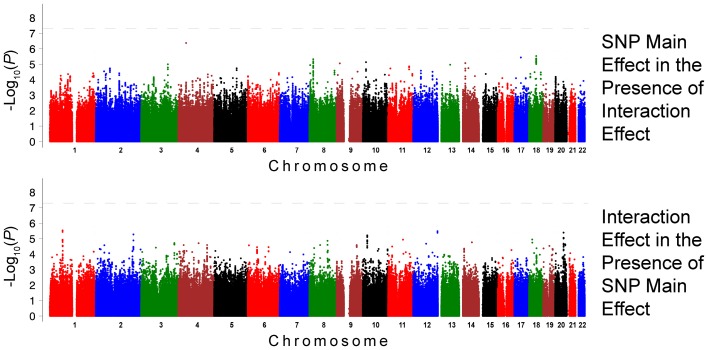
**Association Results for DBP for European Americans: Main and Interaction Effects with Loop Diuretics**.

**Figure 5:**
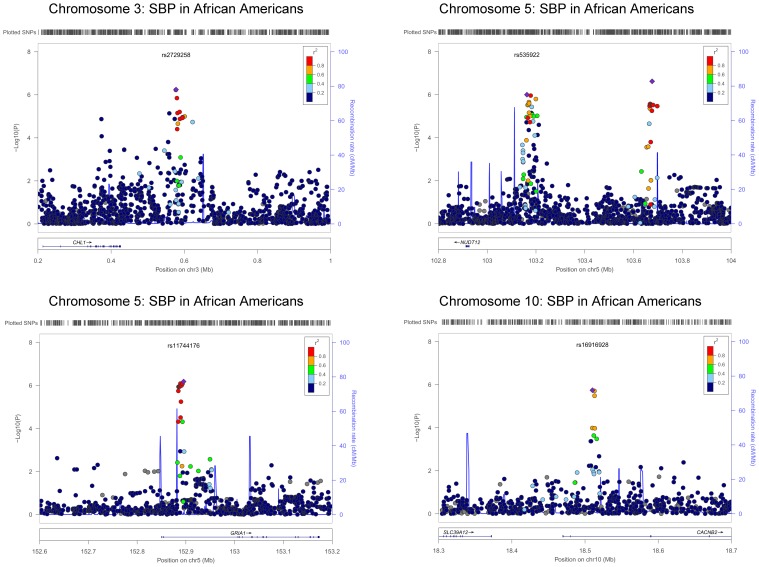
**Regional Plot Results for SBP for African Americans**.

**Figure 6:**
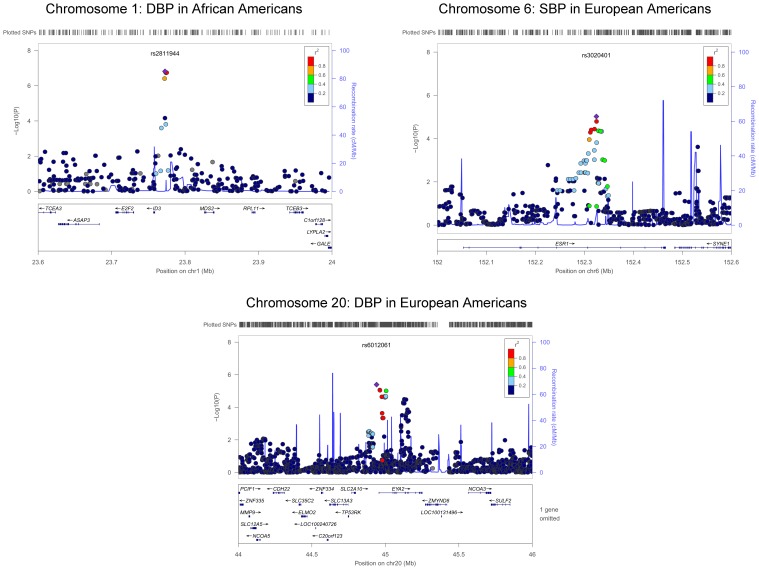
**Regional Plot Results for DBP for European Americans, SBP and DBP for European Americans**.

The QQ plots showed evidence of inflation, in particular for the loop-SNP interaction terms for SBP analyses; Supplement Figure 1 shows the unadjusted *P*-values (black circles) and *P*-values adjusted for the genetic inflation factor lambda (genomic control, blue crosses). Association analysis results are shown in Figures 1–4. Manhattan plots show the adjusted −log_10_(*P*) values for both SBP (Figures 1 and 3) and DBP (Figures 2 and 4) for both races. In each figure, the top Manhattan plot shows the SNP main effect in the presence of the SNP-loop diuretic interaction effect, and the bottom Manhattan plot shows the SNP-loop diuretic interaction in the presence of the SNP main effect. SNPs selected for regional plotting were chosen based on the strong clustering on the Manhattan plots, as well as for the number of SNPs in that region that showed promising levels of significance. Regional plots are shown in Figures 5–7. The top-scoring SNPs for association with gene-medication interactions in BP are shown for AA in Table 2 (*P* < 1 · 10^−6^) and for EA in Table 3 (*P* < 1 · 10^−5^). Online Supplement Tables 2–3 show the 100 most highly ranked SNP-loop diuretic interactions per race. Supplement Table 4 compares these top scoring SNPs from each cohort and phenotype top allow cross-race comparisons.

**Table 2:** **SNPs with Suggestive Association (*P*-value ≤ 1 · 10^−6^) for SBP and DBP in African Americans; Interaction Effect in the Presence of the Main Effect**.

**RS number**	**Chrom**	**Physical position**	**MAF**	**G/I**	**A1**	**A2**	**R^2^**	**Trait**	**SNP main effect**			**SNP-loop interaction effect**			**Nearby genes**	**SNPs per locus**
									**Beta**	***SE***	**Adjusted *P*-value**	**Beta**	***SE***	**Adjusted *P*-value**		
rs2811944	1	23,772,746	0.38	I	T	G	0.75	DBP	−1.10	0.58	5.14 · 10^−2^	11.10	1.96	1.53 · 10^−7^	*ID3-MDS2*	5
rs1633278	1	157,171,831	0.03	I	C	T	0.57	SBP	4.91	3.51	2.12 · 10^−1^	−82.37	13.86	5.59 · 10^−7^	***PYHIN1***	1
rs1432205	2	137,612,734	0.29	I	A	C	0.82	SBP	−0.48	1.09	9.01 · 10^−1^	−19.55	3.88	9.46 · 10^−7^	***THSD7B***	1
rs2729258	3	577,574	0.41	G	C	G	NA	SBP	2.59	0.90	1.73 · 10^−2^	−16.27	2.85	5.71 · 10^−7^	*CHL1-LOC402123*	2
rs6811377	4	41,351,387	0.35	I	A	G	0.94	DBP	1.31	0.53	1.03 · 10^−2^	−9.62	1.78	5.46 · 10^−7^	***LIMCH1***	1
rs535922	5	103,162,869	0.19	I	C	T	0.96	SBP	−1.32	1.14	2.98 · 10^−1^	21.48	3.83	1.00 · 10^−6^	*NUDT12*	1
rs7702688	5	103,676,287	0.06	I	C	A	0.76	SBP	2.62	2.07	2.86 · 10^−1^	−55.33	8.97	2.39 · 10^−7^	*NUDT12*	1
rs11744176	5	152,895,338	0.49	I	T	C	0.99	SBP	0.37	0.89	5.82 · 10^−1^	−16.88	3.08	6.56 · 10^−7^	***GRIA1***	7
rs8040285	15	89,884,905	0.04	I	C	G	0.64	SBP	2.88	3.01	4.14 · 10^−1^	−77.42	13.04	5.55 · 10^−7^	*SV2B-SLCO3A1*	1

Bolded genes represent loci where SNPs are intragenic. A1, allele 1; A2, allele 2; beta, beta coefficient; Chrom, chromosome; DBP, diastolic blood pressure; G/I, genotyped vs. imputed SNP; MAF, minor allele frequency; SBP, systolic blood pressure, SE, standard error of the beta coefficient; SNP, single nucleotide polymorphism.

**Table 3:** **SNPs with Suggestive Association (*P*-value ≤ 1 · 10^−5^) for SBP and DBP in European Americans; Interaction Effect in the Presence of the Main Effect**.

**RS number**	**Chrom**	**Physical position**	**MAF**	**G/I**	**A1**	**A2**	**R^2^**	**Trait**	**SNP main effect**			**SNP-loop interaction effect**			**Nearby genes**	**SNPs per locus**
									**Beta**	***SE***	**Adjusted *P*-value**	**Beta**	***SE***	**Adjusted *P*-value**		
rs3790481	1	68,723,493	0.06	I	C	A	0.83	DBP	0.86	0.96	3.73 · 10^−1^	−31.62	6.47	2.93 · 10^−6^	**DEPDC1**	16
rs6721026	2	200,955,742	0.04	I	A	G	0.79	DBP	−0.23	1.19	8.45 · 10^−1^	−25.38	5.34	5.29 · 10^−6^	**SPATS2L**	2
rs3020401	6	152,324,737	0.35	I	A	G	0.99	SBP	−0.43	0.75	5.64 · 10^−1^	22.77	3.66	9.51 · 10^−6^	**ESR1**	2
rs10764387	10	23,457,184	0.14	I	C	A	0.92	DBP	−0.49	0.61	4.23 · 10^−1^	13.43	2.84	6.15 · 10^−6^	PIP4K2A-ARMC3-	17
															MSRB2-PTF1A	17
rs12303986	12	130,314,492	0.03	G	G	A	NA	DBP	−3.04	1.20	1.11 · 10^−2^	33.16	6.82	3.28 · 10^−6^	GPR133-SFSWAP	2
rs6012061	20	44,940,236	0.04	I	G	A	0.69	DBP	2.06	1.30	1.12 · 10^−1^	−27.26	5.66	4.06 · 10^−6^	**RIT2-EYA2**	12

Bolded genes represent loci where SNPs are intragenic. A1, allele 1; A2, allele 2; beta, beta coefficient; Chrom, chromosome; DBP, diastolic blood pressure; G/I, genotyped vs. imputed SNP; MAF, minor allele frequency; SBP, systolic blood pressure; SE, standard error of the beta coefficient; SNP, single nucleotide polymorphism.

### Race-specific association with SBP and DBP

For the African-American cohort, there were 14 SNPs in 7 loci with suggestive association with SBP (*P* < 1 · 10^−6^; Figure 1); the SNP with the most promising *P*-value was rs7702688 (*P* = 2.39 · 10^−7^) which lies in a relative gene desert ~0.77 Mb downstream of the *NUDT12* gene and is in high pairwise LD with many other SNPs with more modest association located at that locus. Other promising SNPs with suggestive associations with SBP in AA include rs1633278 (*P* = 5.59 · 10^−7^) near *PYHIN1* on chromosome 1, rs2729258 (*P* = 5.71 · 10^−7^) on chromosome 3 which lies ~0.18 Mb downstream of the *CHL1* gene, rs11744176 (*P* = 6.56 · 10^−7^) which is intragenic to *GRIA1* on chromosome 5. Another cluster of SNPs on chromosome 10 with more modest associations with SBP in AA are notable because they are within *CACNB2* (rs16916928; *P* = 1.81 · 10^−6^), a gene previously associated with cardiovascular traits (rs16916928; *P* = 1.81 · 10^−6^). There were 6 SNPs in 2 loci with suggestive association with DBP (*P* < 1 · 10^−6^; Figure 2). The SNP with the lowest *P*-value for DBP in AA was rs2811944 (*P* = 1.53 · 10^−7^), located on chromosome 1 adjacent to the *ID3* gene. Supplement Table 2 shows evidence of strong clustering on chromosomes 3 and 5 (with multiple SNPs in strong LD).

For the European-American cohort, a more permissive *P*-value <1 · 10^−5^ was considered given the relatively lower power due to the fewer number of subjects exposed to loop diuretics (3.9% vs. 7.9% in AA). There was one SNP with a promising association with SBP in EA (rs3020401, *P* = 9.51 · 10^−6^) in *ESR1* on chromosome 6 (Figure 3). A greater number of SNPs, 49 SNPs in 5 loci, showed suggestive associations with DBP in the European-American cohort (*P* < 1 · 10^−5^; Figure 4). Among these SNPs was rs3790481 (*P* = 2.93 · 10^−6^) in *DEPDC1* on chromosome 1, and rs6012061 (*P* = 4.06 · 10^−6^) residing in an intron in *EYA2* on chromosome 20.

No SNP showing strong interaction effect also demonstrated suggestive main effects. It is also notable that a top-100 associated SNP in one cohort/phenotype combination often showed weakened association for the other phenotype in the same cohort (see Supplement Table 4). Corroborative evidence was less commonly noted across race groups. A difference in minor allele frequencies among top SNPs may explain, at least in part, the difference in association across races for some SNPs. For example, rs2729258 on chromosome 3 which has an allele frequency of 0.43 in AA (where *P* = 5.71 · 10^−7^ with SBP), has a frequency of only 0.06 in EA where it showed no association (*P* = 0.64).

### Follow-up sex-stratified analyses

Since genetic variants in *ESR1* have been previously noted to affect BP in a sex-specific manner, the “continuous-covariate” model was repeated in analyses stratified by sex in only the European-American cohort (males: *n* = 613; females: *n* = 618), limiting the regions of interest to the 1 Mb regions surrounding rs3020401. The resulting analyses showed that the association for SBP was greater in European-American men (unadjusted *P* = 4.1 · 10^−7^ for rs3020401) than women (top scoring SNP in region with unadjusted *P* = 2.3 · 10^−4^). The association for DBP in European-American men and women was weaker (unadjusted *P* < 8.2 · 10^−4^ for both).

### Comparison with all subjects model

Secondary analyses were performed in order to test for a possible confounding association between the uses of loop diuretics in subjects with low LVEF. In addition to the primary “Continuous-Covariate” models that analyzed data from subjects with available LVEF as a continuous trait, “All Subjects” models were also performed using the entire cohort (AA 1249 and EA 1267) without consideration of availability or value of LVEF. Compared to our primary results, there were few differences noted in the Manhattan and regional plots for the “All Subjects” analyses in African- and European-Americans cohorts for either SBP or DBP. In general, the overall clustering and trends of association were preserved even if the specific top-scoring signals varied somewhat at any particular locus. In the “All Subjects” model, there were 6 SNPs with suggestive associations clustered on chromosome 17 for SBP in EA that were not seen in the “Continuous-Covariate” model. This locus is 0.15 Mb upstream of the *CA10* gene which encodes a carbonic anhydrase ion channel, the target for a weak diuretic that is more commonly used today to treat glaucoma and altitude sickness.

## Discussion

The relationship between BP and the risk of cardiovascular disease mortality is continuous, consistent, and independent of other risk factors (Lewington et al., 2002). Epidemiologic studies have shown that even modest control of BP on the range of 2–5 mmHg reduction can result in a significant decrease in cardiovascular disease mortality (Cook et al., 1995; Sacks et al., 2001; Whelton et al., 2002). Therefore the identification of SNP-drug interactions for HTN-related traits may identify optimal treatment strategies for patients and regulatory pathways that can be exploited for future drug development and/or personalized treatment of HTN in the general population.

Despite recent GWA studies of HTN-related traits that have included massive sample sizes, <3% of the inter-individual variation in BP has been explained by known (common) variants (Levy et al., 2009; Newton-Cheh et al., 2009; International Consortium for BP GWAS et al., 2011). Since blood pressure-related traits are typically measured during ongoing drug therapy, consideration of SNP-drug interactions may identify additional target loci associated with BP. Herein we report findings from an analysis of GWA data for SNP-loop diuretic medication interactions in systolic and diastolic BPs in the biracial HyperGEN study cohort. We have identified several promising SNPs for both SBP and DBP in the African- and European-American cohorts. It is noteworthy that among the SNPs with the most suggestive interaction effects, none showed equally compelling main effects. It is also notable that SNPs with strong interaction effects in one race cohort rarely showed association in the other. Thus, although none of the reported associations surpassed the stringent Bonferroni-adjusted genome-wide significance threshold (*P* < 5 · 10^−8^), these biologically plausible results nonetheless highlight the importance of evaluating related phenotypes in diverse racial/ethnic cohorts as a strategy to increase the yield for loci relevant to BP regulation and/or antihypertensive drug response and to broaden the generalizability of future therapeutic approaches to multiple patient populations.

Among genes with known associations with cardiovascular traits is *CACNB2* (rs16916928; *P* = 1.81 · 10^−6^) which has drug-SNP interaction effect on SBP in the African-American cohort. The *CACNB2* gene encodes the β 2 subunit of the L-type voltage-gated calcium channel that is widely expressed in cardiovascular tissue. A non-synonymous variant in *CACNB2* has been shown to be responsible for Brugada's syndrome, a heritable sudden cardiac death syndrome (Antzelevitch et al., 2007). This calcium channel is also the target for the anti-hypertensive calcium channel blocker drugs. Variants in *CACNB2* have been associated with HTN traits in numerous, including multiracial, studies (Levy et al., 2009; Ho et al., 2011; International Consortium for BP GWAS et al., 2011; Kato et al., 2011; Lin et al., 2011; Wain et al., 2011). *CACNB2* SNPs were genotyped in the INternational VErapamil SR-Trandolapril STudy-GENEtic substudy (INVEST-GENES), a outcomes study including 5598 hypertensive patients with coronary artery disease who were randomized to either a β-blocker or a calcium channel blocker (Niu et al., 2010). There was a greater risk of adverse outcomes in patients with a rare promoter genotype (G/G, rs2357928) when randomized to the calcium channel blocker vs. the β-blocker, a finding that was consistent in European-, African- and Hispanic-American cohorts. Functional assays showed a significant increase in promoter activity for the rare allele. Since HTN is strongly tied to cardiovascular outcomes, these findings add further evidence of physiologic importance of this gene to cardiovascular traits. In the INVEST-GENES study, only a borderline main effect was detected for rs2357928 in AA; as in our study, rs16916928 was only associated with BP in AA when considered in the context of loop diuretic therapy (Niu et al., 2010). Notably, loop diuretics, by inhibiting the sodium-potassium-chloride channel in the kidney, also increase urinary calcium excretion (Rejnmark et al., 2003); thus, there is biologic plausibility linking diuretic use with SNPs in *CACNB2* in modulating BP regulation.

Among the SNPs with suggestive association for SBP in the European-American cohort was rs3020401 (*P*-value = 9.51 · 10^−6^), which is within the *ESR1*, a gene that encodes the estrogen receptor α. Aside from well-described involvement in gynecologic malignancies and osteoporosis, *ESR1* has also been implicated in mostly candidate gene studies of myocardial infarction, ischemic heart disease risk, in-stent restenosis, sudden cardiac death and age-related changes in left ventricular structure, often with disparate associations in men and women (Ferrero et al., 2003; Schuit et al., 2004; Peter et al., 2007; Aouizerat et al., 2011). There are also examples of variants in *ESR1* interacting with hormone replacement therapy to alter high-density lipoprotein levels and atherosclerosis severity (Herrington et al., 2002; Koivu et al., 2003). A handful of candidate-gene studies have associated genetic variants in ESR1 with BP-related traits. The Victorian Family Heart Study (Ellis et al., 2004) and Framingham Offspring Study (Peter et al., 2005) both identified *ESR1* variants with sex-specific associations with SBP and DBP, respectively, in EA. Furthermore, the GenSalt study, which investigated the genetic determinants of BP response to low- and high-salt diet interventions in Han Chinese, also identified *ESR1* variants associated with DBP in men, but not women (Kelly et al., 2013). Therefore, in order to explore the possibility of a sex-specific interaction between SNPs in ESR1 and loop diuretics, the current study performed additional analyses stratifying the European-American cohort by sex. The associations detected, while less significant due to the smaller sample size, were strongest in men. It is notable that mean ages of the women in both the Victorian Family Heart Study and HyperGEN were 54 and 50 years, respectively; thus, we are not able to determine whether the association between SBP and *ESR1* would be stronger among post-menopausal (estrogen-deficient) women. Although the mechanisms remain unclear, our results suggest that loop diuretics interact with variants in the *ESR1* gene to modulate SBP and that these effects may be further influenced by sex and/or estrogen status.

Among suggestive SNPs, other genes identified have less established roles in cardiovascular disease. The top-scoring drug-SNP interaction for SBP in the African-American cohort is located on chromosome 5 (rs7702688; *P* = 2.39 · 10^−7^) in a relative gene desert ~0.77 Mb downstream of the *NUDT12* gene. *NUDT12* is a Nudix hydrolase, a class of enzymes that regulates levels of circulating diadenosine phosphates (Abdelraheim et al., 2003) which have been shown to have vasoactive properties (Schluter et al., 1994). SNPs in this region have shown marginal associations with BP traits (Gene [database on the Internet] et al., 2013). Also among the more novel findings of this study is an association for SBP in the African-American cohort with SNPs intragenic to *GRIA1* (rs11744176; *P* = 6.56 · 10^−7^) which encodes the alpha-amino-3-hydroxy-5-methyl-4-isoxazole-propionic acid (AMPA)-type glutamate receptor, an ion channel receptor. The binding of the neurotransmitter L-glutamate induces a conformational change allowing the passage of sodium and calcium through the channel, causing depolarization of the neuron. While GWA studies have detected borderline associations with longevity, healthy aging, and sudden cardiac death (Aouizerat et al., 2011; Walter et al., 2011), *GRIA1* has not been identified in other GWA studies of HTN or BP. However, the AMPA receptor has been implicated in the control of fluid ingestion (De Gobbi et al., 2009), a determinant of BP regulation. Perhaps more intriguing, both loop and thiazide diuretics have been shown to potentiate AMPA receptor-dependent ion channel activity by reducing intracellular chloride concentrations in neurons, favoring depolarization (Yamada and Tang, 1993; Sipila et al., 2009). AMPA activation in the hypothalamus increases vasopressin release, a hormone which causes both water retention and vasoconstriction. Thus, there is biologic plausibility that genetic variants in the *GRIA1* gene may modulate the BP response to diuretics (Sladek et al., 1998).

Another interesting finding was identified only in analyses that included all subjects regardless of the availability of the LVEF. SNPs in *CA10* interacted with loop diuretics to modulate SBP in the European-American cohort. Carbonic anhydrases, such as that encoded by *CA10*, function to buffer metabolic sources of acid through reabsorption of bicarbonate, along with sodium and chloride, from the urine (Epstein and Grant, 1977). Synergism between loop diuretics and carbonic anhydrase inhibitors has prompted the use of combination therapy in patients resistant to diuresis (Ellison, 1991). In addition to volume overload, patients with heart failure may experience metabolic acidosis which could favor increased activity of carbonic anhydrases (Reddy et al., 1988); therefore genetic variants that alter the activity of these ion channels may in turn modulate the effectiveness of the loop diuretics to excrete sodium and hence control BP.

SNPs in *SPATS2L* (spermatogenesis-associated serine-rich protein 2-like) have also been associated with both SBP and DBP in a Korean cohort (Hong et al., 2010, 2011). Likewise, SNPs in *EYA2* (eyes absent homolog 2) have been associated with HTN in a Japanese cohort (Kato et al., 2008). Although the mechanisms by which these genes are associated with BP regulation remains elusive, these findings are consistent with the present study where SNPs in both *SPATS2L* and *EYA2* showed suggestive interaction associations with loop diuretics for DBP in AA. The top-scoring SNP for DBP in AA is intragenic to the *ID3* (inhibitor of differentiation 3) gene. This gene encodes a transcription factor that has been found to be protective against atherosclerosis (Doran et al., 2010; Owens et al., 2010); a common loss-of-function SNP in the human *ID3* gene has been linked increased carotid intima-media thickness (Doran et al., 2010), an intermediate trait associated with a variety of cardiovascular outcomes (Lorenz et al., 2006). *CHL1*, a gene associated in this study with SBP in AA, is a known tumor suppressor gene; *CHL1* SNPs have been shown to associate with LDL in interaction models with statin use (Barber et al., 2010).

Given the small proportion of subjects taking loop diuretics in the AA, and in particular the EA cohorts, there was little expectation of finding genome-wide significant interactions due to limited power. Nonetheless, these analyses have identified several genes with promising associations notable for plausible biologic contributions to BP regulation and/or antihypertensive drug response. Given that more permissive thresholds were used to identify suggestive genes in the present study, additional studies are necessary to confirm the findings and to determine potential mechanisms for a role in BP regulation. Our findings may also be limited by not accounting for the use of non-loop diuretics in the comparison group or for the use of combination antihypertensive therapies in the exposure group. This may have further reduced the power. It is also unclear to what degree lack of information on dosage, duration of therapy, and/or medication compliance of loop diuretic therapy may have influenced our results. While not adjusting for LVEF strengthened the association of SNPs in *CA10* which were not identified in the “Continuous-Covariate” model, the prevalence of subjects with an LVEF <45% was low (77 of 1222 African-American and 39 of 1231 European-American subjects), thereby limiting the strength of any conclusions from these analyses. However, since the majority of peaks were common to both the “Continuous-Covariate” and “All Subjects” models, associated loci are unlikely to be the result of bias introduced by the correlation between loop diuretic use and left ventricular systolic dysfunction. In summary, genome-wide interaction analyses that consider the impact of loop diuretic use, including SNP-loop interactions, on BP traits may provide a prioritized set of SNPs and candidate genes worthy of replication and validation in studies of HTN. Studies in more diversified population samples may help identify previously missed variants. In the meantime, these findings make an important contribution to the body of literature aimed at eliminating race disparities through tailored medical therapies.

## Author contributions

Conception and design (Yun Ju Sung, D. C. Rao), administrative support (D. C. Rao), provision of study materials, collection and assembly of data (Steven C. Hunt, Donna K. Arnett, D. C. Rao), data analysis and interpretation (Lisa de las Fuentes, Yun Ju Sung, Karen L. Schwander, Sonia Kalathiveetil, D. C. Rao), manuscript writing (Lisa de las Fuentes, Yun Ju Sung, Karen L. Schwander, Sonia Kalathiveetil), final review and approval of manuscript (all authors). The subject matter of this paper formed part of Sonia Kalathiveetil 's M.S. thesis in the GEMS Program of Washington University.

### Conflict of interest statement

The authors declare that the research was conducted in the absence of any commercial or financial relationships that could be construed as a potential conflict of interest.
