# Supplementary material for: The role of SNP-loop diuretic interactions in hypertension across ethnic groups in HyperGEN
Source: Front Genet. 2013 Dec 25;4:304. doi: 10.3389/fgene.2013.00304 (PMC3872290; doi:10.3389/fgene.2013.00304)
Supplement: Supplementary file 1 [file Presentation1.PDF]

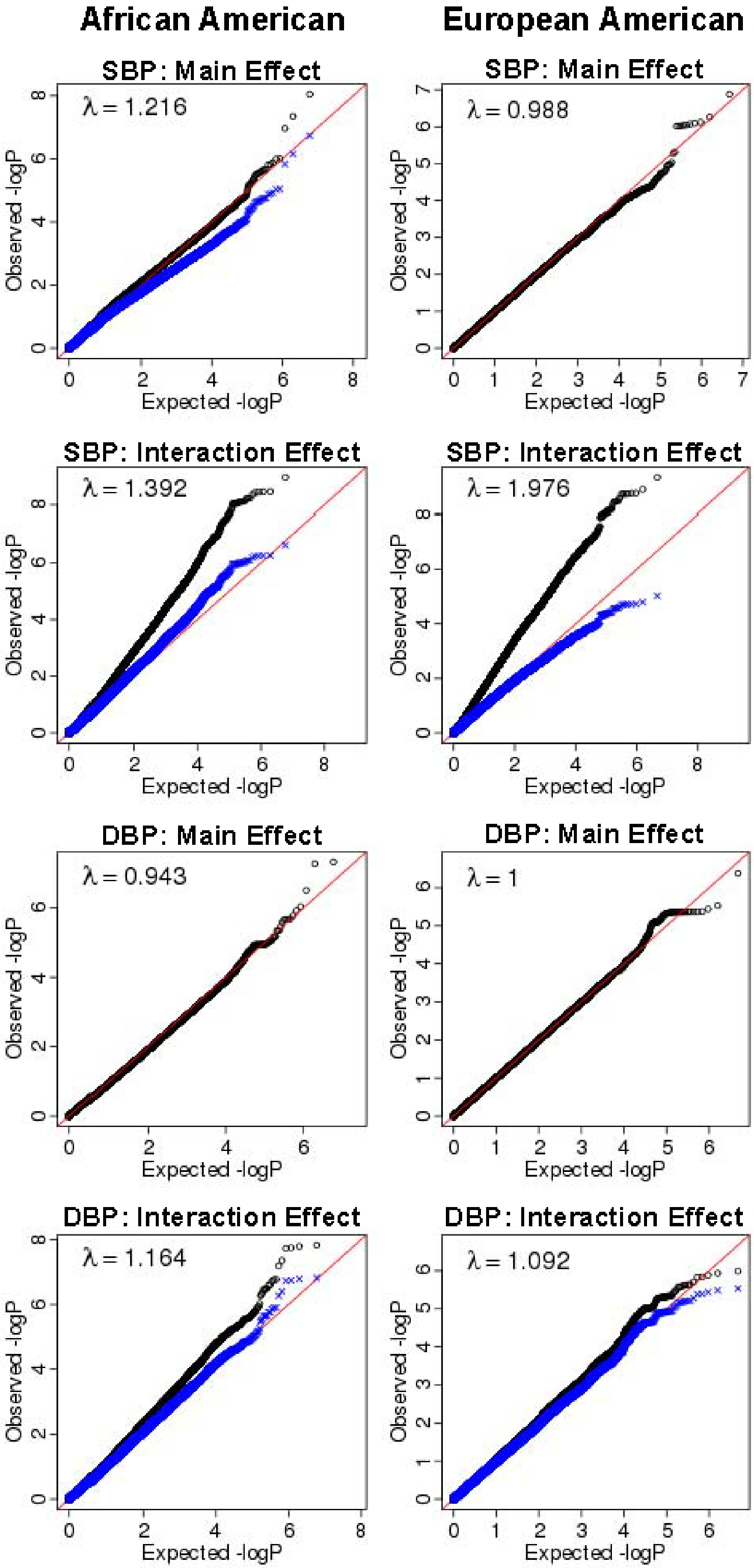

Supplement Figure. QQ plots for the African-American (left panels) and European-American (right panels) cohorts for main and interaction effects. When the unadjusted *P*-values (black circles) demonstrate evidence of inflation, *P*-values adjusted for the genetic inflation factor lambda (genomic control) are also shown (blue crosses).
