# Supplementary material for: The role of SNP-loop diuretic interactions in hypertension across ethnic groups in HyperGEN
Source: Front Genet. 2013 Dec 25;4:304. doi: 10.3389/fgene.2013.00304 (PMC3872290; doi:10.3389/fgene.2013.00304)
Supplement: Supplementary file 2 [file DataSheet1.PDF]

Supplement Table 1. Account of genotyped SNPs remaining following quality control.

Cohort	n	Array	SNPs on Array	Post-QC SNPs	% original panel
EA	1,231	5	443,816	358,327	80.7%
AA	175	5	443,816	392,740	88.5%
AA	1,047	6	909,622	837,134	92.0%
AA	1,222	5.0 & 6.0	914,198 unique SNPs	846,813	92.6%

AA, African American; EA, European American; QC, quality control; SNPs, single nucleotide polymorphisms.
