# Supplementary material for: The role of SNP-loop diuretic interactions in hypertension across ethnic groups in HyperGEN
Source: Front Genet. 2013 Dec 25;4:304. doi: 10.3389/fgene.2013.00304 (PMC3872290; doi:10.3389/fgene.2013.00304)
Supplement: Supplementary file 3 [file DataSheet2.PDF]

## The role of SNP-loop diuretic interactions in hypertension across ethnic groups in HyperGEN

**Supplement Table 2. 100 Top-Ranked SNPs with Suggestive Association for SBP and DBP in African Americans; Interaction Effect in the Presence of the Main Effect.**

| RS Number | Chrom | Physical Postion | MAF | A1 | A2 | $r^2$ | Trait | SNP Main Effect | | | SNP-Loop Interaction Effect | | | *Nearby Genes* | SNPs per Locus |
|---|---|---|---|---|---|---|---|---|---|---|---|---|---|---|---|
| | | | | | | | | Beta | SE | Adjusted *P*-Value | Beta | SE | Adjusted *P*-Value | | |
| rs11579489 | 1 | 15,663,226 | 0.13 | G | A | 0.66 | DBP | -0.89 | 0.87 | 2.91E-01 | 16.06 | 3.21 | 3.44E-06 | *CTRC-ELA2A-* ***CELA2B*** | 1 |
| rs2811944 | 1 | 23,772,746 | 0.38 | T | G | 0.75 | DBP | -1.10 | 0.58 | 5.14E-02 | 11.10 | 1.96 | 1.53E-07 | *ID3-MDS2* | 5 |
| rs1633278 | 1 | 157,171,831 | 0.03 | C | T | 0.57 | SBP | 4.91 | 3.51 | 2.12E-01 | -82.37 | 13.86 | 5.59E-07 | ***PYHIN1*** | 1 |
| rs10177843 | 2 | 136,566,171 | 0.05 | C | T | 0.65 | DBP | 1.97 | 1.32 | 1.25E-01 | -24.90 | 4.76 | 1.26E-06 | *DARS-CXCR4* | 1 |
| rs1432205 | 2 | 137,612,734 | 0.29 | A | C | 0.82 | SBP | -0.48 | 1.09 | 9.01E-01 | -19.55 | 3.88 | 9.46E-07 | ***THSD7B*** | 1 |
| rs1143676 | 2 | 182,103,590 | 0.25 | A | G | 0.99 | DBP | 0.05 | 0.59 | 9.26E-01 | 9.59 | 1.88 | 2.27E-06 | *MIR4437-* ***ITGA4*** | 2 |
| rs2729258 | 3 | 577,574 | 0.41 | C | G | NA | SBP | 2.59 | 0.90 | 1.73E-02 | -16.27 | 2.85 | 5.71E-07 | *CHL1-LOC402123* | 2 |
| rs2729207 | 3 | 579,868 | 0.41 | A | T | NA | SBP | 2.50 | 0.90 | 2.25E-02 | -15.76 | 2.87 | 1.42E-06 | *CHL1-LOC402123* | 1 |
| rs2729243 | 3 | 581,433 | 0.45 | A | G | 0.96 | SBP | 2.35 | 0.91 | 4.71E-02 | -14.76 | 2.85 | 7.02E-06 | *CHL1-LOC402123* | 1 |
| rs2729148 | 3 | 587,273 | 0.45 | A | G | 0.92 | SBP | 2.17 | 0.93 | 7.09E-02 | -15.28 | 2.95 | 6.23E-06 | *CHL1-LOC402123* | 1 |
| rs17786807 | 3 | 2,337,468 | 0.12 | G | C | NA | DBP | -0.51 | 0.76 | 4.87E-01 | 13.02 | 2.49 | 1.29E-06 | ***CNTN4*** | 2 |
| rs6441770 | 3 | 43,377,250 | 0.09 | T | C | 0.94 | SBP | 0.95 | 1.55 | 4.27E-01 | -32.53 | 6.10 | 3.81E-06 | *SNRK-ANO10* | 2 |
| rs1383019 | 3 | 141,597,174 | 0.15 | T | G | 0.89 | SBP | 1.85 | 1.31 | 1.66E-01 | -26.74 | 4.95 | 6.03E-06 | ***CLSTN2*** | 1 |
| rs16877398 | 4 | 25,278,862 | 0.10 | G | A | NA | SBP | 1.90 | 1.52 | 2.03E-01 | -26.44 | 5.17 | 5.15E-06 | ***SLC34A2*** | 2 |
| rs1863309 | 4 | 35,246,435 | 0.08 | A | C | 0.82 | DBP | -2.25 | 1.01 | 2.12E-02 | 19.29 | 3.93 | 5.56E-06 | *ARAP2* | 1 |
| rs1371293 | 4 | 41,348,655 | 0.42 | A | G | 0.92 | DBP | 1.00 | 0.52 | 4.66E-02 | -8.64 | 1.66 | 1.40E-06 | ***LIMCH1*** | 2 |
| rs7721823 | 5 | 103,178,198 | 0.20 | A | G | 0.98 | SBP | -2.16 | 1.12 | 8.53E-02 | 20.61 | 3.67 | 1.10E-06 | *NUDT12* | 10 |
| rs7702688 | 5 | 103,676,287 | 0.06 | C | A | 0.76 | SBP | 2.62 | 2.07 | 2.86E-01 | -55.33 | 8.97 | 2.39E-07 | *NUDT12* | 13 |
| rs9327483 | 5 | 128,044,164 | 0.10 | G | A | 0.85 | SBP | -1.00 | 1.59 | 5.77E-01 | 25.67 | 5.08 | 5.60E-06 | *FBN2-SLC27A6* | 1 |
| rs10037138 | 5 | 147,596,566 | 0.48 | T | A | 0.94 | SBP | -0.79 | 0.91 | 4.18E-01 | 16.03 | 3.01 | 5.32E-06 | *SPINK6-SPINK5L7* | 7 |
| rs11744176 | 5 | 152,895,338 | 0.49 | T | C | 0.99 | SBP | 0.37 | 0.89 | 5.82E-01 | -16.88 | 3.08 | 6.56E-07 | ***GRIA1*** | 18 |
| rs984184 | 7 | 11,264,568 | 0.32 | C | G | 0.65 | DBP | -0.68 | 0.65 | 2.81E-01 | 10.95 | 2.25 | 6.64E-06 | *PHF14-THSD7A* | 1 |
| rs11974360 | 7 | 30,337,311 | 0.38 | A | G | 0.98 | SBP | 1.55 | 0.91 | 6.13E-02 | -16.65 | 3.18 | 5.63E-06 | *MIR550A1- MIR550B1-* ***ZNRF2*** | 1 |
| rs2643800 | 9 | 27,647,713 | 0.17 | T | G | 0.93 | DBP | -0.23 | 0.66 | 7.24E-01 | 11.91 | 2.31 | 1.76E-06 | *MOB3B-IFNK-LINGO2* | 2 |
| rs16916928 | 10 | 18,510,159 | 0.30 | G | A | NA | SBP | 2.09 | 0.99 | 6.91E-02 | -18.00 | 3.43 | 1.81E-06 | *SLC39A12-* ***CACNB2*** | 3 |
| rs4492852 | 11 | 50,499,436 | 0.49 | G | C | 0.95 | SBP | 0.86 | 0.91 | 4.57E-01 | -15.45 | 2.88 | 3.93E-06 | *LOC646813-OR4A6P* | 1 |
| rs4417255 | 11 | 51,061,717 | 0.44 | A | G | NA | SBP | -0.62 | 0.90 | 5.12E-01 | 15.40 | 2.92 | 4.47E-06 | *LOC646813-OR4A6P* | 1 |
| rs2186641 | 11 | 51,163,494 | 0.46 | T | C | 0.91 | SBP | -0.34 | 0.93 | 7.70E-01 | 15.98 | 3.08 | 6.68E-06 | *LOC646813-OR4A6P* | 1 |
| rs554731 | 11 | 51,244,515 | 0.47 | C | T | 0.96 | SBP | -0.49 | 0.90 | 6.61E-01 | 15.65 | 2.87 | 2.14E-06 | *LOC646813-OR4A6P* | 5 |
| rs17655864 | 11 | 110,336,581 | 0.06 | T | C | 0.79 | DBP | 3.12 | 1.20 | 7.67E-03 | -22.10 | 4.34 | 2.31E-06 | *ARHGAP20-MIR4491-POU2AF1* | 1 |
| rs1946519 | 11 | 111,540,717 | 0.35 | C | A | 0.95 | DBP | 0.62 | 0.53 | 2.24E-01 | -9.19 | 1.83 | 3.16E-06 | *IL18-* ***TEX12*** *-BCO2* | 1 |
| rs9568642 | 13 | 51,090,690 | 0.13 | G | A | 0.82 | SBP | -1.15 | 1.48 | 5.78E-01 | 22.47 | 4.81 | 3.17E-06 | *MIR4703-* ***WDFY2-*** *DHRS12* | 1 |
| rs12430231 | 13 | 64,347,888 | 0.14 | G | A | 0.86 | SBP | 3.50 | 1.39 | 1.98E-02 | -27.01 | 4.92 | 2.12E-06 | *NFYAP-LGMN2P* | 1 |
| rs8040285 | 15 | 89,884,905 | 0.04 | C | G | 0.64 | SBP | 2.88 | 3.01 | 4.14E-01 | -77.42 | 13.04 | 5.55E-07 | *SV2B-SLCO3A1* | 1 |
| rs12964689 | 18 | 19,370,996 | 0.49 | A | G | 0.97 | SBP | 2.80 | 0.90 | 3.29E-03 | -16.70 | 3.17 | 1.86E-06 | *C18orf8-* ***NPC1*** | 2 |
| rs504368 | 18 | 53,933,770 | 0.17 | T | C | 0.39 | SBP | 1.20 | 1.89 | 6.10E-01 | -30.50 | 5.85 | 6.89E-06 | ***NEDD4L*** | 1 |
| rs8094402 | 18 | 72,836,437 | 0.10 | A | G | 0.75 | DBP | -0.35 | 0.94 | 7.00E-01 | -16.01 | 3.30 | 6.72E-06 | ***MBP*** | 1 |

**Bolded** genes represent loci where SNPs are intragenic. SNPs with r2 = NA are genotyped. A1, allele 1; A2, allele 2; beta, beta coefficient; Chrom, chromosome; DBP, diastolic blood pressure; G/I, genotyped vs. imputed SNP; MAF, minor allele frequency; SBP, systolic blood pressure, SE, standard error of the beta coefficient; SNP, single nucleotide
