# Supplementary material for: The role of SNP-loop diuretic interactions in hypertension across ethnic groups in HyperGEN
Source: Front Genet. 2013 Dec 25;4:304. doi: 10.3389/fgene.2013.00304 (PMC3872290; doi:10.3389/fgene.2013.00304)
Supplement: Supplementary file 4 [file DataSheet3.PDF]

The role of SNP-loop diuretic interactions in hypertension across ethnic groups in HyperGEN

Supplement Table 3. 100 Top-Ranked SNPs with Suggestive Association for SBP and DBP in European Americans; Interaction Effect in the Presence of the Main Effect.

RS Number	Chrom	Physical Postion	MAF	A1	A2	R^2	Trait	SNP Main Effect			SNP-Loop Interaction Effect			*Nearby Genes*	SNPs per Locus
								Beta	SE	Adjusted *P*-Value	Beta	SE	Adjusted *P*-Value		
rs3790481	1	68,723,493	0.06	C	A	0.83	DBP	0.86	0.96	3.73E-01	-31.62	6.47	2.93E-06	***DEPDC1***	16
rs7534917	1	68,835,848	0.05	G	C	0.99	DBP	0.86	0.96	3.74E-01	-29.48	6.55	1.65E-05	*DEPDC1-LOC101927220*	1
rs6721026	2	200,955,742	0.04	A	G	0.79	DBP	-0.23	1.19	8.45E-01	-25.38	5.34	5.29E-06	***SPATS2L***	2
rs6800441	3	177,560,665	0.06	A	C	NA	DBP	-0.06	0.85	9.41E-01	30.20	6.76	1.93E-05	*NAALADL2-MIR4789-TBL1XR1*	17
rs4696666	4	8,070,347	0.12	G	A	0.68	SBP	2.23	1.39	1.05E-01	-29.96	5.04	2.36E-05	*AFAP1-**ABLIM2**-MIR95*	1
rs2047030	4	28,289,533	0.03	C	G	0.77	DBP	-0.88	1.36	5.17E-01	-31.26	7.11	2.60E-05	*MIR4275*	1
rs427152	4	108,244,429	0.07	G	A	0.95	DBP	0.20	0.82	8.08E-01	-20.12	4.51	1.97E-05	*DKK2*	1
rs4696138	4	153,738,440	0.38	T	G	0.79	DBP	-0.13	0.47	7.77E-01	-9.06	2.06	2.58E-05	*FBXW7-MIR3140-MIR4453-TMEM154*	1
rs3020401	6	152,324,737	0.35	A	G	0.99	SBP	-0.43	0.75	5.64E-01	22.77	3.66	9.51E-06	***ESR1***	2
rs6474079	8	57,786,137	0.17	T	C	0.77	DBP	1.33	0.62	3.25E-02	-11.66	2.65	2.59E-05	*PENK-IMPAD1*	1
rs2586998	8	97,175,303	0.03	C	A	0.78	DBP	0.37	1.28	7.71E-01	-25.89	5.70	1.37E-05	*GDF6-MTERFD1*	2
rs10816298	9	107,494,543	0.06	C	G	1.00	DBP	1.02	0.87	2.44E-01	-17.13	3.90	2.60E-05	*FKTN-TAL2-TMEM38B*	5
rs4469535	9	121,616,941	0.26	T	G	0.99	SBP	1.70	0.84	4.27E-02	-22.71	3.78	1.89E-05	*BRINP1-MEGF9*	7
rs10764387	10	23,457,184	0.14	C	A	0.92	DBP	-0.49	0.61	4.23E-01	13.43	2.84	6.15E-06	*PIP4K2A-ARMC3-MSRB2-PTF1A*	17
rs17311507	11	79,870,481	0.07	G	C	0.44	DBP	-1.71	1.16	1.41E-01	37.08	8.09	1.15E-05	*LOC101928964*	1
rs17814434	12	69,369,774	0.04	A	G	NA	DBP	1.08	1.04	2.99E-01	-24.81	5.58	2.12E-05	*PTPRB-P**TPRR***	1
rs12303986	12	130,314,492	0.03	G	A	NA	DBP	-3.04	1.20	1.11E-02	33.16	6.82	3.28E-06	*GPR133-SFSWAP*	2
rs10137893	14	68,142,631	0.14	T	C	0.49	DBP	-1.09	0.84	1.92E-01	-16.36	3.64	1.72E-05	***RAD51B**-ZFP36L1-ACTN1*	1
rs11660409	18	17,179,348	0.04	G	A	0.76	DBP	0.87	1.16	4.54E-01	-25.45	5.55	1.12E-05	***GREB1L**-ESCO1-ABHD3*	1
rs11662661	18	17,502,690	0.05	T	C	0.77	DBP	0.07	1.11	9.51E-01	-24.68	5.50	1.78E-05	*GREB1L-ESCO1-**ABHD3***	1
rs6012061	20	44,940,236	0.04	G	A	0.69	DBP	2.06	1.30	1.12E-01	-27.26	5.66	4.06E-06	***RIT2-EYA2***	12
rs6064344	20	54,155,639	0.02	A	G	0.94	DBP	0.37	1.53	8.08E-01	-31.24	7.06	2.31E-05	*CBLN4-MC3R*	7

Bolded genes represent loci where SNPs are intragenic. SNPs with r2 = NA are genotyped. A1, allele 1; A2, allele 2; beta, beta coefficient; Chrom, chromosome; DBP, diastolic blood pressure; G/I, genotyped vs. imputed SNP; MAF, minor allele frequency; SBP, systolic blood pressure, SE, standard error of the beta coefficient; SNP, single nucleotide polymorphism.
