# Supplementary material for: The role of SNP-loop diuretic interactions in hypertension across ethnic groups in HyperGEN
Source: Front Genet. 2013 Dec 25;4:304. doi: 10.3389/fgene.2013.00304 (PMC3872290; doi:10.3389/fgene.2013.00304)
Supplement: Supplementary file 5 [file DataSheet4.PDF]

The role of SNP-loop diuretic interactions in hypertension across ethnic groups in HyperGEN

Supplement Table 4. Cross-Race Comparisons of 100 Top-Ranked SNPs with Suggestive Association for SBP and DBP in African Americans and European Americans; Interaction Effect in the Presence of the Main Effect.

RS Number	Chrom	Physical Postion	Trait	African American								European American							
				MAF	r^2	SNP Main			SNP-Loop Interaction Effect			MAF	r^2	SNP Main			SNP-Loop Interaction Effect		
						Beta	SE	Adjusted *P*-Value	Beta	SE	Adjusted *P*-Value			Beta	SE	Adjusted *P*-Value	Beta	SE	Adjusted *P*-Value
rs11579489	1	15,663,226	SBP	0.06	0.66	3.35	2.08	1.05E-01	-11.85	13.49	5.32E-01	0.13	0.50	-1.60	1.56	4.17E-01	18.98	5.84	5.01E-03
rs11579489	1	15,663,226	DBP	0.06	0.66	2.02	1.16	8.22E-02	-5.24	7.51	5.04E-01	0.13	0.50	-0.89	0.87	2.91E-01	16.06	3.21	3.44E-06
rs2742951	1	23,772,132	SBP	0.47	0.73	-0.20	0.76	7.87E-01	1.73	3.22	7.02E-01	0.44	0.91	-2.42	1.05	4.94E-02	15.99	3.50	1.50E-04
rs2742951	1	23,772,132	DBP	0.47	0.73	-0.04	0.43	9.16E-01	1.65	1.79	3.77E-01	0.44	0.91	-0.79	0.58	1.62E-01	10.77	1.97	3.94E-07
rs2811944	1	23,772,746	SBP	0.45	0.75	-0.07	0.81	9.33E-01	2.65	3.55	5.96E-01	0.38	0.81	-2.77	1.06	2.63E-02	16.77	3.50	4.87E-05
rs2811944	1	23,772,746	DBP	0.45	0.75	0.12	0.45	7.86E-01	2.04	1.97	3.23E-01	0.38	0.81	-1.10	0.58	5.14E-02	11.10	1.96	1.53E-07
rs2811945	1	23,773,130	SBP	0.45	0.76	-0.07	0.81	9.32E-01	2.67	3.56	5.94E-01	0.37	0.80	-2.79	1.05	2.47E-02	16.38	3.48	6.88E-05
rs2811945	1	23,773,130	DBP	0.45	0.76	0.12	0.45	7.85E-01	2.05	1.98	3.22E-01	0.37	0.80	-1.18	0.58	3.69E-02	11.02	1.95	1.65E-07
rs2742955	1	23,774,189	SBP	0.45	0.76	-0.07	0.82	9.32E-01	2.75	3.60	5.87E-01	0.36	0.79	-2.96	1.06	1.79E-02	16.40	3.52	7.92E-05
rs2742955	1	23,774,189	DBP	0.45	0.76	0.13	0.46	7.82E-01	2.09	2.01	3.18E-01	0.36	0.79	-1.18	0.58	3.68E-02	11.10	1.97	1.87E-07
rs2811946	1	23,775,003	SBP	0.47	0.74	-0.30	0.86	7.28E-01	1.54	3.88	7.78E-01	0.38	0.71	-2.78	1.06	2.64E-02	17.14	3.52	3.66E-05
rs2811946	1	23,775,003	DBP	0.47	0.74	0.08	0.48	8.70E-01	1.68	2.16	4.56E-01	0.38	0.71	-1.02	0.58	7.10E-02	11.11	1.97	1.81E-07
rs3790481	1	68,723,493	SBP	0.06	0.98	-1.74	1.72	3.10E-01	-53.89	11.62	9.71E-04	0.14	0.83	-0.56	1.31	7.29E-01	2.48	4.51	7.02E-01
rs3790481	1	68,723,493	DBP	0.06	0.98	0.86	0.96	3.73E-01	-31.62	6.47	2.93E-06	0.14	0.83	-0.05	0.72	9.47E-01	2.92	2.48	2.74E-01
rs1886298	1	68,774,903	SBP	0.06	0.80	-1.44	1.69	3.91E-01	-51.18	11.93	2.28E-03	0.04	0.86	2.66	2.57	3.79E-01	5.00	8.48	5.87E-01
rs1886298	1	68,774,903	DBP	0.06	0.80	0.90	0.94	3.40E-01	-32.15	6.65	3.65E-06	0.04	0.86	1.80	1.46	2.05E-01	3.53	4.62	4.78E-01
rs17772203	2	102,482,292	SBP	0.06	NA	0.95	1.59	5.47E-01	7.27	6.54	4.29E-01	0.09	NA	-3.39	1.58	4.79E-02	28.71	5.59	9.76E-06
rs17772203	2	102,482,292	DBP	0.06	NA	-0.31	0.89	7.25E-01	3.15	3.64	4.07E-01	0.50	NA	-1.54	0.87	6.77E-02	11.19	3.03	6.32E-04
rs1835804	2	137,592,803	SBP	0.40	0.93	-0.79	0.84	3.46E-01	1.67	3.95	7.63E-01	0.28	0.77	-0.85	1.03	6.16E-01	-16.80	3.66	9.35E-06
rs1835804	2	137,592,803	DBP	0.40	0.93	0.06	0.47	9.00E-01	3.56	2.19	1.20E-01	0.28	0.77	-1.03	0.57	6.08E-02	-6.08	2.06	6.23E-03
rs1432205	2	137,612,734	SBP	0.40	0.82	-0.78	0.85	3.59E-01	1.52	3.98	7.86E-01	0.29	0.76	-0.48	1.09	9.01E-01	-19.55	3.88	9.46E-07
rs1432205	2	137,612,734	DBP	0.40	0.82	0.07	0.48	8.84E-01	3.53	2.21	1.26E-01	0.29	0.76	-0.68	0.60	2.45E-01	-7.27	2.20	2.17E-03
rs3770104	2	182,102,390	SBP	0.33	0.99	0.83	0.77	2.80E-01	-1.18	3.19	7.93E-01	0.25	0.99	-0.31	1.08	8.13E-01	11.28	3.42	5.56E-03
rs3770104	2	182,102,390	DBP	0.33	0.99	0.26	0.43	5.40E-01	0.33	1.78	8.58E-01	0.25	0.99	0.08	0.59	8.93E-01	9.52	1.87	2.32E-06
rs1143676	2	182,103,590	SBP	0.33	0.99	0.82	0.77	2.80E-01	-1.16	3.18	7.95E-01	0.25	0.99	-0.35	1.07	7.80E-01	11.48	3.44	4.98E-03
rs1143676	2	182,103,590	DBP	0.33	0.99	0.26	0.43	5.41E-01	0.32	1.77	8.62E-01	0.25	0.99	0.05	0.59	9.26E-01	9.59	1.88	2.27E-06
rs6721026	2	200,955,742	SBP	0.04	0.82	0.27	2.14	9.01E-01	-37.40	9.59	5.53E-03	0.01	0.79	2.35	4.44	5.04E-01	2.12	12.12	8.99E-01
rs6721026	2	200,955,742	DBP	0.04	0.82	-0.23	1.19	8.45E-01	-25.38	5.34	5.29E-06	0.01	0.79	0.91	2.49	7.05E-01	6.51	6.62	3.62E-01
rs9873368	3	559,082	SBP	0.07	0.90	1.39	1.52	3.57E-01	3.89	5.68	6.26E-01	0.29	0.83	3.02	1.03	8.12E-03	-18.25	3.47	7.24E-06
rs9873368	3	559,082	DBP	0.07	0.90	0.47	0.85	5.76E-01	2.82	3.16	3.93E-01	0.29	0.83	1.25	0.57	2.55E-02	-4.51	1.91	2.86E-02
rs9811400	3	576,918	SBP	0.06	NA	1.87	1.51	2.11E-01	3.59	5.67	6.52E-01	0.43	NA	1.98	0.89	6.07E-02	-15.67	2.87	5.91E-07
rs9811400	3	576,918	DBP	0.06	NA	0.94	0.84	2.63E-01	2.70	3.15	4.12E-01	0.50	NA	0.90	0.49	6.06E-02	-5.91	1.58	5.35E-04
rs2729258	3	577,574	SBP	0.06	NA	1.68	1.51	2.64E-01	3.77	5.67	6.37E-01	0.41	NA	2.59	0.90	1.73E-02	-16.27	2.85	5.71E-07
rs2729258	3	577,574	DBP	0.06	NA	0.88	0.84	2.95E-01	2.76	3.15	4.03E-01	0.50	NA	1.17	0.50	1.60E-02	-5.49	1.56	1.09E-03
rs2729207	3	579,868	SBP	0.06	NA	1.67	1.55	2.76E-01	3.77	5.68	6.37E-01	0.41	NA	2.50	0.90	2.25E-02	-15.76	2.87	1.42E-06

Supplement Table 4. Cross-Race Comparisons of 100 Top-Ranked SNPs with Suggestive Association for SBP and DBP in African Americans and European Americans; Interaction Effect in the Presence of the Main Effect.

RS Number	Chrom	Physical Postion	Trait	African American								European American							
				MAF	r^2	SNP Main			SNP-Loop Interaction Effect			MAF	r^2	SNP Main			SNP-Loop Interaction Effect		
						Beta	SE	Adjusted *P* -Value	Beta	SE	Adjusted *P* -Value			Beta	SE	Adjusted *P* -Value	Beta	SE	Adjusted *P* -Value
rs2729207	3	579,868	DBP	0.06	NA	0.81	0.86	3.45E-01	2.82	3.16	3.93E-01	0.50	NA	1.16	0.50	1.70E-02	-5.41	1.57	1.38E-03
rs2729243	3	581,433	SBP	0.05	0.96	1.69	1.66	3.06E-01	4.83	6.08	5.72E-01	0.45	1.00	2.35	0.91	4.71E-02	-14.76	2.85	7.02E-06
rs2729243	3	581,433	DBP	0.05	0.96	0.61	0.93	5.13E-01	2.46	3.38	4.86E-01	0.45	1.00	1.08	0.50	2.71E-02	-5.71	1.55	6.49E-04
rs2729148	3	587,273	SBP	0.07	0.92	1.52	1.50	3.07E-01	4.80	5.54	5.38E-01	0.45	0.89	2.17	0.93	7.09E-02	-15.28	2.95	6.23E-06
rs2729148	3	587,273	DBP	0.07	0.92	0.77	0.83	3.56E-01	2.78	3.08	3.89E-01	0.45	0.89	1.03	0.51	3.97E-02	-5.29	1.61	2.34E-03
rs3967750	3	2,337,071	SBP	0.24	NA	1.13	0.80	1.55E-01	4.24	3.96	4.46E-01	0.12	NA	-0.22	1.35	8.47E-01	19.33	4.68	3.76E-04
rs3967750	3	2,337,071	DBP	0.24	NA	-0.32	0.45	4.71E-01	3.65	2.20	1.13E-01	0.12	NA	-0.58	0.75	4.22E-01	12.88	2.52	2.18E-06
rs17786807	3	2,337,468	SBP	0.24	NA	1.26	0.86	1.40E-01	2.87	4.50	6.51E-01	0.12	NA	-0.29	1.37	8.15E-01	20.20	4.62	1.69E-04
rs17786807	3	2,337,468	DBP	0.24	NA	-0.21	0.48	6.60E-01	3.55	2.50	1.75E-01	0.12	NA	-0.51	0.76	4.87E-01	13.02	2.49	1.29E-06
rs1383019	3	141,597,174	SBP	0.06	0.89	-4.56	1.73	7.83E-03	12.29	8.88	3.25E-01	0.15	0.78	1.85	1.31	1.66E-01	-26.74	4.95	6.03E-06
rs1383019	3	141,597,174	DBP	0.06	0.89	-2.24	0.96	2.00E-02	7.54	4.94	1.44E-01	0.15	0.78	0.88	0.72	2.12E-01	-7.48	2.75	1.19E-02
rs16877398	4	25,278,862	SBP	0.05	NA	-0.47	1.69	7.78E-01	11.76	12.11	4.89E-01	0.10	NA	1.90	1.52	2.03E-01	-26.44	5.17	5.15E-06
rs16877398	4	25,278,862	DBP	0.05	NA	0.10	0.95	9.19E-01	13.30	6.74	5.90E-02	0.10	NA	-0.25	0.84	7.63E-01	-9.86	2.83	1.22E-03
rs3815464	4	25,280,141	SBP	0.05	0.88	0.54	1.76	7.57E-01	-13.87	12.76	4.39E-01	0.11	0.95	2.17	1.52	1.50E-01	-26.47	5.22	6.60E-06
rs3815464	4	25,280,141	DBP	0.05	0.88	-0.05	0.98	9.63E-01	-14.82	7.10	4.59E-02	0.11	0.95	-0.22	0.84	7.91E-01	-9.59	2.85	1.81E-03
rs1863309	4	35,246,435	SBP	0.29	0.82	-1.17	0.86	1.72E-01	3.82	4.42	5.38E-01	0.08	0.84	-2.12	1.83	2.90E-01	16.21	6.98	3.00E-02
rs1863309	4	35,246,435	DBP	0.29	0.82	-0.71	0.48	1.40E-01	-1.56	2.46	5.44E-01	0.08	0.84	-2.25	1.01	2.12E-02	19.29	3.93	5.56E-06
rs6836342	4	35,294,985	SBP	0.30	0.86	-1.33	0.89	1.31E-01	4.64	4.36	4.49E-01	0.08	0.79	-2.62	1.77	1.94E-01	16.77	6.99	2.62E-02
rs6836342	4	35,294,985	DBP	0.30	0.86	-1.00	0.50	4.46E-02	-2.05	2.42	4.18E-01	0.08	0.79	-1.81	0.97	5.62E-02	18.80	3.94	9.75E-06
rs1371293	4	41,348,655	SBP	0.38	0.92	0.45	0.85	5.94E-01	2.83	4.29	6.38E-01	0.42	0.81	1.56	0.94	1.41E-01	-13.34	3.01	5.62E-05
rs1371293	4	41,348,655	DBP	0.38	0.92	0.89	0.47	6.01E-02	0.99	2.38	6.92E-01	0.42	0.81	1.00	0.52	4.66E-02	-8.64	1.66	1.40E-06
rs6811377	4	41,351,387	SBP	0.42	0.94	0.38	0.80	6.39E-01	2.12	4.24	7.23E-01	0.35	0.89	1.74	0.96	1.41E-01	-15.05	3.21	1.93E-05
rs6811377	4	41,351,387	DBP	0.42	0.94	0.71	0.45	1.12E-01	0.23	2.36	9.26E-01	0.35	0.89	1.31	0.53	1.03E-02	-9.62	1.78	5.46E-07
rs535922	5	103,162,869	SBP	0.47	0.96	-0.91	0.74	2.14E-01	-3.48	3.51	4.80E-01	0.19	0.99	-1.32	1.14	2.98E-01	21.48	3.83	1.00E-06
rs535922	5	103,162,869	DBP	0.47	0.96	-0.08	0.41	8.39E-01	-1.43	1.95	4.82E-01	0.19	0.99	0.01	0.63	9.84E-01	7.44	2.11	1.06E-03
rs557434	5	103,165,604	SBP	0.49	0.96	-0.72	0.74	3.29E-01	-3.69	3.51	4.55E-01	0.22	0.97	-1.52	1.09	2.19E-01	19.78	3.77	3.04E-06
rs557434	5	103,165,604	DBP	0.49	0.96	-0.02	0.42	9.53E-01	-1.54	1.95	4.52E-01	0.22	0.97	0.02	0.60	9.78E-01	6.92	2.08	2.00E-03
rs1990902	5	103,169,679	SBP	0.48	0.96	-0.73	0.74	3.20E-01	-3.66	3.51	4.58E-01	0.24	0.99	-0.75	1.06	5.43E-01	18.07	3.60	2.87E-06
rs1990902	5	103,169,679	DBP	0.48	0.96	-0.03	0.41	9.34E-01	-1.48	1.95	4.67E-01	0.24	0.99	-0.02	0.58	9.71E-01	6.65	2.04	2.51E-03
rs1422094	5	103,171,054	SBP	0.48	0.97	-0.69	0.74	3.44E-01	-3.69	3.51	4.55E-01	0.22	1.00	-1.58	1.08	1.93E-01	18.24	3.58	2.20E-06
rs1422094	5	103,171,054	DBP	0.48	0.97	-0.02	0.41	9.52E-01	-1.49	1.95	4.64E-01	0.22	1.00	-0.20	0.59	7.31E-01	6.99	2.02	1.35E-03
rs1422095	5	103,171,431	SBP	0.48	0.97	-0.69	0.74	3.46E-01	-3.69	3.51	4.55E-01	0.24	1.00	-1.15	1.05	3.46E-01	17.22	3.56	6.90E-06
rs1422095	5	103,171,431	DBP	0.48	0.97	-0.02	0.41	9.53E-01	-1.49	1.95	4.64E-01	0.24	1.00	-0.10	0.58	8.53E-01	6.31	2.01	3.63E-03
rs2193997	5	103,171,744	SBP	0.48	0.98	-0.69	0.74	3.47E-01	-3.69	3.51	4.54E-01	0.22	1.00	-1.60	1.08	1.87E-01	17.97	3.54	2.51E-06
rs2193997	5	103,171,744	DBP	0.48	0.98	-0.02	0.41	9.54E-01	-1.49	1.95	4.64E-01	0.22	1.00	-0.14	0.59	8.02E-01	6.86	2.00	1.44E-03

The role of SNP-loop diuretic interactions in hypertension across ethnic groups in HyperGEN

Supplement Table 4. Cross-Race Comparisons of 100 Top-Ranked SNPs with Suggestive Association for SBP and DBP in African Americans and European Americans; Interaction Effect in the Presence of the Main Effect.

				African American								European American							
						SNP Main			SNP-Loop Interaction Effect					SNP Main			SNP-Loop Interaction Effect		
RS Number	Chrom	Physical Postion	Trait	MAF	r^2	Beta	SE	Adjusted *P*-Value	Beta	SE	Adjusted *P*-Value	MAF	r^2	Beta	SE	Adjusted *P*-Value	Beta	SE	Adjusted *P*-Value
rs2161206	5	103,175,667	SBP	0.48	0.99	-0.69	0.74	3.49E-01	-3.69	3.51	4.54E-01	0.19	0.99	-1.90	1.14	1.39E-01	18.79	3.69	9.95E-06
rs2161206	5	103,175,667	DBP	0.48	0.99	-0.02	0.41	9.53E-01	-1.49	1.95	4.64E-01	0.19	0.99	0.07	0.63	9.10E-01	6.50	2.03	2.95E-03
rs7721823	5	103,178,198	SBP	0.48	0.98	-0.69	0.74	3.49E-01	-3.69	3.51	4.55E-01	0.20	0.99	-2.16	1.12	8.53E-02	20.61	3.67	1.10E-06
rs7721823	5	103,178,198	DBP	0.48	0.98	-0.03	0.41	9.51E-01	-1.49	1.95	4.64E-01	0.20	0.99	-0.12	0.62	8.36E-01	7.13	2.01	1.03E-03
rs10900869	5	103,181,097	SBP	0.48	0.90	-0.69	0.74	3.52E-01	-3.69	3.53	4.58E-01	0.13	0.98	-1.20	1.42	4.41E-01	21.16	4.12	7.01E-06
rs10900869	5	103,181,097	DBP	0.48	0.90	-0.03	0.41	9.51E-01	-1.52	1.97	4.58E-01	0.13	0.98	0.63	0.78	4.06E-01	6.78	2.26	5.51E-03
rs1592831	5	103,182,607	SBP	0.47	NA	-0.54	0.75	4.66E-01	-3.69	3.54	4.59E-01	0.20	0.96	-2.02	1.11	9.62E-02	18.43	3.46	3.70E-06
rs1592831	5	103,182,607	DBP	0.47	NA	0.10	0.42	8.06E-01	-1.67	1.97	4.18E-01	0.20	0.96	0.33	0.62	5.78E-01	5.34	1.89	8.75E-03
rs12719525	5	103,183,554	SBP	0.47	0.91	-0.54	0.75	4.66E-01	-3.68	3.54	4.60E-01	0.14	0.96	-1.10	1.35	4.46E-01	20.12	3.93	7.69E-06
rs12719525	5	103,183,554	DBP	0.47	0.91	0.10	0.42	8.07E-01	-1.67	1.97	4.18E-01	0.14	0.96	0.98	0.74	1.75E-01	5.01	2.15	3.12E-02
rs10069578	5	103,184,521	SBP	0.47	0.99	-0.54	0.75	4.67E-01	-3.68	3.55	4.60E-01	0.20	0.96	-2.12	1.14	9.81E-02	18.71	3.64	8.48E-06
rs10069578	5	103,184,521	DBP	0.47	0.99	0.10	0.42	8.06E-01	-1.67	1.97	4.17E-01	0.20	0.96	0.33	0.63	5.90E-01	5.37	2.00	1.26E-02
rs6887457	5	103,199,464	SBP	0.48	0.96	0.41	0.77	5.94E-01	0.58	3.93	9.16E-01	0.19	0.91	2.03	1.17	1.12E-01	-20.26	3.67	1.59E-06
rs6887457	5	103,199,464	DBP	0.48	0.96	-0.22	0.43	6.07E-01	1.03	2.19	6.53E-01	0.19	0.91	-0.08	0.65	8.94E-01	-5.95	2.01	6.04E-03
rs1363248	5	103,204,973	SBP	0.49	0.92	0.15	0.79	8.45E-01	0.67	4.00	9.06E-01	0.24	0.85	1.83	1.08	1.28E-01	-18.58	3.63	9.58E-06
rs1363248	5	103,204,973	DBP	0.49	0.92	-0.27	0.44	5.40E-01	1.20	2.23	6.05E-01	0.24	0.85	-0.02	0.60	9.79E-01	-5.34	1.99	1.30E-02
rs6873252	5	103,665,930	SBP	0.03	0.94	-1.91	2.24	3.90E-01	6.10	16.64	7.94E-01	0.07	0.95	0.59	1.74	7.79E-01	-44.73	8.15	3.38E-06
rs6873252	5	103,665,930	DBP	0.03	0.94	-0.14	1.25	9.13E-01	15.51	9.25	1.08E-01	0.07	0.95	0.47	0.97	6.17E-01	-10.25	4.39	3.05E-02
rs13360898	5	103,667,083	SBP	0.03	0.92	-1.91	2.24	3.91E-01	6.08	16.64	7.95E-01	0.08	0.95	0.44	1.73	8.17E-01	-42.03	7.77	4.40E-06
rs13360898	5	103,667,083	DBP	0.03	0.92	-0.14	1.25	9.14E-01	15.51	9.25	1.08E-01	0.08	0.95	0.50	0.96	5.87E-01	-9.48	4.18	3.54E-02
rs10066860	5	103,667,203	SBP	0.03	0.96	-1.90	2.24	3.92E-01	6.08	16.64	7.95E-01	0.07	0.95	0.58	1.74	7.81E-01	-44.65	8.06	2.75E-06
rs10066860	5	103,667,203	DBP	0.03	0.96	-0.13	1.25	9.14E-01	15.51	9.25	1.08E-01	0.07	0.95	0.46	0.96	6.24E-01	-10.27	4.34	2.83E-02
rs10051458	5	103,667,478	SBP	0.03	0.96	-1.90	2.24	3.93E-01	6.01	16.64	7.97E-01	0.07	0.95	0.58	1.74	7.81E-01	-44.64	8.05	2.73E-06
rs10051458	5	103,667,478	DBP	0.03	0.96	-0.13	1.25	9.15E-01	15.49	9.25	1.09E-01	0.07	0.95	0.46	0.96	6.24E-01	-10.27	4.34	2.82E-02
rs10053410	5	103,668,244	SBP	0.03	0.96	-1.87	2.24	4.00E-01	5.99	16.65	7.98E-01	0.07	0.95	0.58	1.74	7.82E-01	-44.63	8.05	2.68E-06
rs10053410	5	103,668,244	DBP	0.03	0.96	-0.13	1.25	9.19E-01	15.50	9.26	1.09E-01	0.07	0.95	0.46	0.96	6.25E-01	-10.27	4.33	2.81E-02
rs7718281	5	103,670,306	SBP	0.03	0.97	-1.85	2.24	4.07E-01	5.91	16.66	8.01E-01	0.07	0.95	0.60	1.73	7.74E-01	-44.37	8.03	2.91E-06
rs7718281	5	103,670,306	DBP	0.03	0.97	-0.12	1.25	9.24E-01	15.50	9.26	1.09E-01	0.07	0.95	0.48	0.96	6.06E-01	-10.24	4.32	2.81E-02
rs13357162	5	103,671,977	SBP	0.03	0.96	-1.82	2.24	4.14E-01	5.82	16.66	8.04E-01	0.07	0.95	0.43	1.72	8.38E-01	-44.42	8.05	2.98E-06
rs13357162	5	103,671,977	DBP	0.03	0.96	-0.11	1.25	9.28E-01	15.46	9.26	1.10E-01	0.07	0.95	0.48	0.95	6.06E-01	-10.33	4.33	2.71E-02
rs10057012	5	103,675,212	SBP	0.03	0.75	-1.82	2.24	4.14E-01	5.81	16.66	8.04E-01	0.07	0.94	2.59	1.97	2.67E-01	-35.75	7.13	5.59E-06
rs10057012	5	103,675,212	DBP	0.03	0.75	-0.12	1.25	9.26E-01	15.43	9.26	1.11E-01	0.07	0.94	1.68	1.09	1.13E-01	-13.12	4.04	2.61E-03
rs7702688	5	103,676,287	SBP	0.03	0.76	-1.63	2.13	4.40E-01	5.19	16.66	8.25E-01	0.06	0.92	2.62	2.07	2.86E-01	-55.33	8.97	2.39E-07
rs7702688	5	103,676,287	DBP	0.03	0.76	0.48	1.19	6.85E-01	14.84	9.26	1.25E-01	0.06	0.92	1.90	1.15	8.79E-02	-14.57	4.84	5.25E-03
rs7722681	5	103,676,705	SBP	0.03	0.96	-1.83	2.24	4.13E-01	5.75	16.67	8.06E-01	0.07	0.94	0.41	1.72	8.45E-01	-44.47	8.06	3.00E-06

The role of SNP-loop diuretic interactions in hypertension across ethnic groups in HyperGEN

Supplement Table 4. Cross-Race Comparisons of 100 Top-Ranked SNPs with Suggestive Association for SBP and DBP in African Americans and European Americans; Interaction Effect in the Presence of the Main Effect.

RS Number	Chrom	Physical Postion	Trait	African American								European American							
						SNP Main			SNP-Loop Interaction Effect					SNP Main			SNP-Loop Interaction Effect		
				MAF	r^2	Beta	SE	Adjusted *P*-Value	Beta	SE	Adjusted *P*-Value	MAF	r^2	Beta	SE	Adjusted *P*-Value	Beta	SE	Adjusted *P*-Value
rs7722681	5	103,676,705	DBP	0.03	0.96	-0.12	1.25	9.24E-01	15.38	9.26	1.12E-01	0.07	0.94	0.47	0.95	6.14E-01	-10.34	4.34	2.72E-02
rs13328089	5	103,678,935	SBP	0.03	0.96	-1.83	2.24	4.13E-01	5.73	16.67	8.07E-01	0.07	0.94	0.41	1.72	8.46E-01	-44.48	8.06	3.01E-06
rs13328089	5	103,678,935	DBP	0.03	0.96	-0.12	1.25	9.23E-01	15.36	9.26	1.12E-01	0.07	0.94	0.47	0.95	6.14E-01	-10.34	4.34	2.72E-02
rs9327898	5	103,680,070	SBP	0.03	0.96	-1.83	2.24	4.13E-01	5.73	16.67	8.07E-01	0.07	0.94	0.41	1.72	8.47E-01	-44.47	8.06	3.01E-06
rs9327898	5	103,680,070	DBP	0.03	0.96	-0.12	1.25	9.23E-01	15.36	9.26	1.12E-01	0.07	0.94	0.47	0.95	6.15E-01	-10.34	4.34	2.72E-02
rs7702100	5	103,697,274	SBP	0.03	0.90	-1.86	2.26	4.08E-01	5.30	16.75	8.22E-01	0.07	0.91	0.32	1.75	8.89E-01	-45.82	8.35	3.40E-06
rs7702100	5	103,697,274	DBP	0.03	0.90	-0.16	1.26	9.02E-01	14.88	9.31	1.26E-01	0.07	0.91	0.38	0.97	6.87E-01	-10.40	4.50	3.20E-02
rs6894351	5	112,073,031	SBP	0.45	NA	-1.22	0.76	1.07E-01	0.99	3.83	8.54E-01	0.49	NA	-0.14	0.90	7.25E-01	16.02	3.15	9.02E-06
rs6894351	5	112,073,031	DBP	0.45	NA	-0.42	0.43	3.26E-01	-2.13	2.13	3.38E-01	0.50	NA	-0.19	0.49	6.95E-01	5.59	1.71	2.40E-03
rs1465910	5	112,073,730	SBP	0.45	0.99	-1.22	0.76	1.07E-01	0.99	3.83	8.54E-01	0.49	1.00	0.14	0.90	7.21E-01	-16.13	3.18	9.93E-06
rs1465910	5	112,073,730	DBP	0.45	0.99	-0.42	0.43	3.25E-01	-2.13	2.13	3.38E-01	0.49	1.00	0.13	0.49	7.83E-01	-5.54	1.73	2.98E-03
rs10073062	5	127,887,473	SBP	0.31	0.85	-0.23	0.78	7.65E-01	-3.50	3.63	4.93E-01	0.34	0.99	1.28	1.04	3.95E-01	-19.91	3.78	9.13E-06
rs10073062	5	127,887,473	DBP	0.31	0.85	-0.14	0.44	7.45E-01	-1.38	2.02	5.12E-01	0.34	0.99	0.43	0.57	4.41E-01	-9.52	2.06	1.86E-05
rs9327483	5	128,044,164	SBP	0.01	0.85	-0.57	3.41	8.67E-01	6.93	13.16	7.08E-01	0.10	0.91	-1.00	1.59	5.77E-01	25.67	5.08	5.60E-06
rs9327483	5	128,044,164	DBP	0.01	0.85	2.59	1.91	1.74E-01	-1.08	7.31	8.88E-01	0.10	0.91	-0.24	0.88	7.76E-01	12.27	2.82	5.66E-05
rs10038416	5	147,573,424	SBP	0.07	0.95	2.52	1.52	9.53E-02	-1.64	6.03	8.47E-01	0.47	0.96	-0.50	0.91	6.21E-01	15.89	3.05	9.53E-06
rs10038416	5	147,573,424	DBP	0.07	0.95	0.98	0.85	2.49E-01	-1.68	3.36	6.33E-01	0.47	0.96	-0.65	0.50	1.82E-01	7.47	1.68	3.98E-05
rs10075545	5	147,586,050	SBP	0.07	1.00	2.55	1.49	8.53E-02	-2.07	5.97	8.05E-01	0.47	0.99	-0.48	0.89	6.31E-01	15.68	2.97	6.94E-06
rs10075545	5	147,586,050	DBP	0.07	1.00	1.00	0.83	2.32E-01	-1.65	3.32	6.34E-01	0.47	0.99	-0.58	0.49	2.23E-01	7.34	1.64	3.44E-05
rs6876753	5	147,588,160	SBP	0.07	1.00	2.56	1.49	8.38E-02	-2.14	5.96	7.98E-01	0.47	1.00	-0.48	0.89	6.34E-01	15.66	2.97	6.87E-06
rs6876753	5	147,588,160	DBP	0.07	1.00	1.00	0.83	2.29E-01	-1.65	3.32	6.35E-01	0.47	1.00	-0.58	0.49	2.24E-01	7.33	1.64	3.45E-05
rs12517179	5	147,588,821	SBP	0.07	NA	-2.64	1.48	7.25E-02	2.87	5.88	7.28E-01	0.47	NA	0.50	0.89	6.19E-01	-15.68	2.97	6.71E-06
rs12517179	5	147,588,821	DBP	0.07	NA	-1.05	0.83	2.04E-01	1.60	3.27	6.39E-01	0.50	NA	0.62	0.49	1.94E-01	-7.36	1.64	3.17E-05
rs1432693	5	147,589,329	SBP	0.06	NA	-2.56	1.51	8.67E-02	-1.54	6.11	8.57E-01	0.47	NA	-0.48	0.89	6.34E-01	15.66	2.97	6.87E-06
rs1432693	5	147,589,329	DBP	0.06	NA	-1.11	0.84	1.86E-01	2.15	3.40	5.45E-01	0.50	NA	-0.58	0.49	2.25E-01	7.33	1.64	3.45E-05
rs10037138	5	147,596,566	SBP	0.07	0.94	2.55	1.49	8.44E-02	-2.14	5.96	7.98E-01	0.48	1.00	-0.79	0.91	4.18E-01	16.03	3.01	5.32E-06
rs10037138	5	147,596,566	DBP	0.07	0.94	1.00	0.83	2.29E-01	-1.65	3.32	6.35E-01	0.48	1.00	-0.71	0.50	1.46E-01	6.75	1.67	1.80E-04
rs2895730	5	147,596,582	SBP	0.07	1.00	2.55	1.49	8.44E-02	-2.14	5.96	7.98E-01	0.47	1.00	-0.51	0.89	6.13E-01	15.66	2.96	6.80E-06
rs2895730	5	147,596,582	DBP	0.07	1.00	1.00	0.83	2.29E-01	-1.65	3.32	6.35E-01	0.47	1.00	-0.56	0.49	2.40E-01	7.31	1.64	3.62E-05
rs10055415	5	147,598,299	SBP	0.07	1.00	2.55	1.49	8.44E-02	-2.15	5.96	7.98E-01	0.47	1.00	-0.51	0.89	6.15E-01	15.65	2.96	6.91E-06
rs10055415	5	147,598,299	DBP	0.07	1.00	1.00	0.83	2.29E-01	-1.65	3.32	6.35E-01	0.47	1.00	-0.56	0.49	2.41E-01	7.31	1.64	3.64E-05
rs1159205	5	147,599,522	SBP	0.07	1.00	2.55	1.49	8.46E-02	-2.15	5.96	7.97E-01	0.47	1.00	-0.48	0.89	6.33E-01	15.50	2.96	8.19E-06
rs1159205	5	147,599,522	DBP	0.07	1.00	1.00	0.83	2.28E-01	-1.65	3.32	6.35E-01	0.47	1.00	-0.55	0.49	2.53E-01	7.27	1.64	3.90E-05
rs10058023	5	147,600,033	SBP	0.07	1.00	2.55	1.49	8.45E-02	-2.15	5.96	7.97E-01	0.47	1.00	-0.48	0.89	6.37E-01	15.47	2.96	8.51E-06
rs10058023	5	147,600,033	DBP	0.07	1.00	1.00	0.83	2.28E-01	-1.65	3.32	6.35E-01	0.47	1.00	-0.54	0.49	2.55E-01	7.26	1.64	3.97E-05

The role of SNP-loop diuretic interactions in hypertension across ethnic groups in HyperGEN

Supplement Table 4. Cross-Race Comparisons of 100 Top-Ranked SNPs with Suggestive Association for SBP and DBP in African Americans and European Americans; Interaction Effect in the Presence of the Main Effect.

RS Number	Chrom	Physical Postion	Trait	African American								European American							
				MAF	r^2	SNP Main			SNP-Loop Interaction Effect			MAF	r^2	SNP Main			SNP-Loop Interaction Effect		
						Beta	SE	Adjusted *P*-Value	Beta	SE	Adjusted *P*-Value			Beta	SE	Adjusted *P*-Value	Beta	SE	Adjusted *P*-Value
rs545098	5	152,884,481	SBP	0.33	NA	-0.33	0.79	6.71E-01	-5.16	4.26	3.89E-01	0.49	NA	-0.41	0.89	5.45E-01	16.13	3.07	1.79E-06
rs545098	5	152,884,481	DBP	0.33	NA	-0.37	0.44	3.97E-01	-2.01	2.37	4.17E-01	0.50	NA	-0.30	0.49	5.25E-01	5.08	1.71	5.95E-03
rs541518	5	152,884,874	SBP	0.33	NA	-0.19	0.79	8.09E-01	-5.28	4.26	3.78E-01	0.49	NA	-0.21	0.89	6.99E-01	16.28	3.05	1.15E-06
rs541518	5	152,884,874	DBP	0.33	NA	-0.24	0.44	5.84E-01	-2.11	2.37	3.93E-01	0.50	NA	-0.22	0.49	6.49E-01	5.29	1.70	3.85E-03
rs517589	5	152,885,199	SBP	0.33	1.00	-0.21	0.79	7.92E-01	-5.27	4.26	3.79E-01	0.49	1.00	0.21	0.89	7.01E-01	-16.29	3.05	1.14E-06
rs517589	5	152,885,199	DBP	0.33	1.00	-0.22	0.44	6.13E-01	-2.13	2.37	3.90E-01	0.49	1.00	0.22	0.49	6.49E-01	-5.30	1.70	3.83E-03
rs515709	5	152,885,414	SBP	0.33	1.00	-0.21	0.79	7.91E-01	-5.26	4.26	3.79E-01	0.49	1.00	0.21	0.89	7.01E-01	-16.30	3.05	1.13E-06
rs515709	5	152,885,414	DBP	0.33	1.00	-0.22	0.44	6.12E-01	-2.13	2.37	3.90E-01	0.49	1.00	0.22	0.49	6.49E-01	-5.31	1.70	3.78E-03
rs495703	5	152,886,033	SBP	0.33	1.00	-0.21	0.79	7.90E-01	-5.26	4.26	3.79E-01	0.49	1.00	0.21	0.89	7.01E-01	-16.32	3.05	1.12E-06
rs495703	5	152,886,033	DBP	0.33	1.00	-0.22	0.44	6.11E-01	-2.13	2.37	3.90E-01	0.49	1.00	0.22	0.49	6.49E-01	-5.32	1.70	3.74E-03
rs480726	5	152,886,920	SBP	0.33	1.00	-0.21	0.79	7.89E-01	-5.26	4.26	3.80E-01	0.49	1.00	0.19	0.89	7.14E-01	-16.31	3.05	1.13E-06
rs480726	5	152,886,920	DBP	0.33	1.00	-0.22	0.44	6.10E-01	-2.13	2.37	3.90E-01	0.49	1.00	0.21	0.49	6.64E-01	-5.31	1.70	3.77E-03
rs479946	5	152,886,965	SBP	0.33	1.00	-0.21	0.79	7.88E-01	-5.26	4.26	3.80E-01	0.49	1.00	0.21	0.89	7.01E-01	-16.35	3.05	1.09E-06
rs479946	5	152,886,965	DBP	0.33	1.00	-0.22	0.44	6.09E-01	-2.13	2.37	3.90E-01	0.49	1.00	0.22	0.49	6.49E-01	-5.33	1.70	3.66E-03
rs479767	5	152,887,029	SBP	0.33	1.00	-0.21	0.79	7.86E-01	-5.26	4.26	3.80E-01	0.49	1.00	0.21	0.89	7.01E-01	-16.36	3.05	1.08E-06
rs479767	5	152,887,029	DBP	0.33	1.00	-0.23	0.44	6.07E-01	-2.13	2.37	3.91E-01	0.49	1.00	0.22	0.49	6.49E-01	-5.34	1.70	3.64E-03
rs559294	5	152,888,050	SBP	0.33	1.00	-0.21	0.79	7.85E-01	-5.26	4.26	3.80E-01	0.49	1.00	0.18	0.89	7.19E-01	-16.37	3.06	1.09E-06
rs559294	5	152,888,050	DBP	0.33	1.00	-0.23	0.44	6.07E-01	-2.13	2.37	3.91E-01	0.49	1.00	0.20	0.49	6.72E-01	-5.35	1.70	3.62E-03
rs517931	5	152,888,735	SBP	0.33	0.97	-0.22	0.79	7.82E-01	-5.26	4.26	3.80E-01	0.44	1.00	-0.17	0.91	1.00E+00	-15.50	2.96	8.11E-07
rs517931	5	152,888,735	DBP	0.33	0.97	-0.23	0.44	6.04E-01	-2.12	2.37	3.91E-01	0.44	1.00	-0.15	0.50	7.62E-01	-5.70	1.66	1.49E-03
rs489572	5	152,889,546	SBP	0.33	1.00	-0.22	0.79	7.77E-01	-5.25	4.26	3.80E-01	0.49	1.00	0.21	0.89	7.01E-01	-16.44	3.06	1.01E-06
rs489572	5	152,889,546	DBP	0.33	1.00	-0.23	0.44	6.01E-01	-2.12	2.37	3.91E-01	0.49	1.00	0.22	0.49	6.49E-01	-5.39	1.71	3.39E-03
rs488443	5	152,889,716	SBP	0.32	0.99	-0.22	0.80	7.80E-01	-6.05	4.33	3.20E-01	0.49	0.96	0.20	0.89	7.09E-01	-16.55	3.08	9.98E-07
rs488443	5	152,889,716	DBP	0.32	0.99	-0.22	0.45	6.23E-01	-2.18	2.41	3.87E-01	0.49	0.96	0.21	0.49	6.63E-01	-5.46	1.72	3.22E-03
rs487362	5	152,889,877	SBP	0.33	0.99	-0.23	0.79	7.73E-01	-5.25	4.26	3.81E-01	0.49	1.00	0.19	0.89	7.11E-01	-15.27	3.06	5.66E-06
rs487362	5	152,889,877	DBP	0.33	0.99	-0.23	0.44	5.97E-01	-2.12	2.37	3.92E-01	0.49	1.00	0.19	0.49	6.93E-01	-4.77	1.71	9.67E-03
rs4958659	5	152,891,096	SBP	0.33	0.99	-0.23	0.79	7.72E-01	-5.25	4.26	3.81E-01	0.49	1.00	0.17	0.89	7.32E-01	-16.50	3.07	9.95E-07
rs4958659	5	152,891,096	DBP	0.33	0.99	-0.23	0.44	5.96E-01	-2.12	2.37	3.92E-01	0.49	1.00	0.19	0.49	6.94E-01	-5.44	1.71	3.24E-03
rs4958660	5	152,891,106	SBP	0.33	0.99	-0.23	0.79	7.71E-01	-5.25	4.26	3.81E-01	0.49	1.00	0.17	0.89	7.32E-01	-16.51	3.07	9.92E-07
rs4958660	5	152,891,106	DBP	0.33	0.99	-0.23	0.44	5.95E-01	-2.12	2.37	3.92E-01	0.49	1.00	0.19	0.49	6.95E-01	-5.44	1.71	3.23E-03
rs726877	5	152,892,862	SBP	0.33	1.00	-0.23	0.79	7.68E-01	-5.24	4.26	3.82E-01	0.49	1.00	0.22	0.89	6.92E-01	-16.66	3.08	8.39E-07
rs726877	5	152,892,862	DBP	0.33	1.00	-0.24	0.44	5.93E-01	-2.12	2.37	3.92E-01	0.49	1.00	0.22	0.49	6.42E-01	-5.57	1.72	2.62E-03
rs726876	5	152,893,041	SBP	0.33	1.00	-0.24	0.79	7.64E-01	-5.23	4.27	3.83E-01	0.49	1.00	0.22	0.89	6.90E-01	-16.68	3.08	8.24E-07
rs726876	5	152,893,041	DBP	0.33	1.00	-0.24	0.44	5.91E-01	-2.12	2.37	3.93E-01	0.49	1.00	0.22	0.49	6.41E-01	-5.59	1.72	2.54E-03
rs11744176	5	152,895,338	SBP	0.31	0.99	-0.47	0.81	5.62E-01	-8.15	4.50	1.97E-01	0.49	0.97	0.37	0.89	5.82E-01	-16.88	3.08	6.56E-07

Supplement Table 4. Cross-Race Comparisons of 100 Top-Ranked SNPs with Suggestive Association for SBP and DBP in African Americans and European Americans; Interaction Effect in the Presence of the Main Effect.

RS Number	Chrom	Physical Postion	Trait	African American								European American							
				MAF	r^2	SNP Main			SNP-Loop Interaction Effect			MAF	r^2	SNP Main			SNP-Loop Interaction Effect		
						Beta	SE	Adjusted *P*-Value	Beta	SE	Adjusted *P*-Value			Beta	SE	Adjusted *P*-Value	Beta	SE	Adjusted *P*-Value
rs11744176	5	152,895,338	DBP	0.31	0.99	-0.40	0.45	3.77E-01	-3.96	2.50	1.30E-01	0.49	0.97	0.27	0.49	5.75E-01	-5.67	1.72	2.26E-03
rs3020401	6	152,324,737	SBP	0.35	0.98	-0.43	0.75	5.64E-01	22.77	3.66	9.51E-06	0.33	0.99	1.44	0.94	1.95E-01	-7.80	3.08	3.76E-02
rs3020401	6	152,324,737	DBP	0.35	0.98	-0.25	0.42	5.46E-01	6.01	2.04	4.71E-03	0.33	0.99	1.06	0.52	3.60E-02	-2.26	1.75	2.30E-01
rs984184	7	11,264,568	SBP	0.44	0.65	-0.96	0.90	2.85E-01	3.09	4.54	6.29E-01	0.32	0.68	-0.62	1.17	7.17E-01	17.99	4.10	2.32E-04
rs984184	7	11,264,568	DBP	0.44	0.65	-0.13	0.50	7.97E-01	3.02	2.53	2.52E-01	0.32	0.68	-0.68	0.65	2.81E-01	10.95	2.25	6.64E-06
rs2643800	9	27,647,713	SBP	0.37	0.93	-0.55	0.79	4.84E-01	3.75	3.62	4.61E-01	0.17	0.95	-1.18	1.20	4.49E-01	18.44	4.23	1.78E-04
rs2643800	9	27,647,713	DBP	0.37	0.93	-0.33	0.44	4.46E-01	0.25	2.01	9.05E-01	0.17	0.95	-0.23	0.66	7.24E-01	11.91	2.31	1.76E-06
rs2783014	9	27,648,306	SBP	0.37	0.93	-0.55	0.79	4.84E-01	3.74	3.60	4.59E-01	0.17	0.96	-1.17	1.20	4.49E-01	18.44	4.23	1.77E-04
rs2783014	9	27,648,306	DBP	0.37	0.93	-0.33	0.44	4.47E-01	0.25	2.00	9.06E-01	0.17	0.96	-0.23	0.66	7.25E-01	11.90	2.31	1.76E-06
rs16916928	10	18,510,159	SBP	0.28	NA	-1.36	0.78	7.77E-02	-0.47	3.40	9.22E-01	0.30	NA	2.09	0.99	6.91E-02	-18.00	3.43	1.81E-06
rs16916928	10	18,510,159	DBP	0.28	NA	-0.45	0.43	2.97E-01	1.65	1.89	4.02E-01	0.30	NA	0.13	0.55	8.02E-01	-4.67	1.91	2.37E-02
rs1757207	10	18,512,867	SBP	0.28	0.96	1.35	0.82	9.66E-02	0.09	3.68	9.85E-01	0.33	0.99	-1.90	0.98	9.16E-02	18.10	3.45	1.94E-06
rs1757207	10	18,512,867	DBP	0.28	0.96	0.66	0.46	1.50E-01	-1.49	2.05	4.84E-01	0.33	0.99	-0.09	0.54	8.65E-01	5.03	1.92	1.52E-02
rs1779226	10	18,512,983	SBP	0.24	0.94	1.21	0.87	1.63E-01	-0.60	4.02	9.15E-01	0.32	0.99	-1.72	1.00	1.34E-01	19.08	3.56	3.27E-06
rs1779226	10	18,512,983	DBP	0.24	0.94	0.55	0.49	2.58E-01	-0.29	2.24	9.02E-01	0.32	0.99	-0.04	0.55	9.41E-01	5.19	1.94	1.34E-02
rs11013298	10	23,454,501	SBP	0.12	0.90	0.62	1.18	6.00E-01	18.25	5.38	1.58E-02	0.49	0.85	0.92	0.93	5.61E-01	-1.63	3.35	7.71E-01
rs11013298	10	23,454,501	DBP	0.12	0.90	-0.46	0.66	4.91E-01	14.11	2.99	6.44E-06	0.49	0.85	0.01	0.51	9.87E-01	2.12	1.84	2.87E-01
rs10764387	10	23,457,184	SBP	0.14	0.93	0.58	1.09	5.88E-01	17.60	5.11	1.43E-02	0.44	0.92	1.12	0.91	4.03E-01	-4.68	3.59	3.05E-01
rs10764387	10	23,457,184	DBP	0.14	0.93	-0.49	0.61	4.23E-01	13.43	2.84	6.15E-06	0.44	0.92	0.30	0.50	5.34E-01	1.86	1.98	3.84E-01
rs1925700	10	23,459,528	SBP	0.13	NA	0.66	1.08	5.40E-01	17.40	5.11	1.54E-02	0.43	0.95	1.55	0.82	8.62E-02	-3.92	2.80	2.67E-01
rs1925700	10	23,459,528	DBP	0.13	NA	-0.45	0.60	4.60E-01	13.41	2.84	6.32E-06	0.50	0.95	0.44	0.45	3.17E-01	0.40	1.55	8.09E-01
rs10734043	10	23,461,938	SBP	0.13	0.93	0.66	1.08	5.39E-01	17.39	5.11	1.54E-02	0.42	0.96	1.45	0.92	2.38E-01	-4.94	3.57	2.61E-01
rs10734043	10	23,461,938	DBP	0.13	0.93	-0.45	0.60	4.61E-01	13.40	2.84	6.34E-06	0.42	0.96	0.43	0.51	3.85E-01	2.44	1.97	2.50E-01
rs2057599	10	23,471,624	SBP	0.13	NA	0.69	1.07	5.15E-01	17.02	5.07	1.69E-02	0.41	0.99	-0.75	0.89	4.70E-01	4.21	3.41	2.90E-01
rs2057599	10	23,471,624	DBP	0.13	NA	-0.39	0.60	5.11E-01	13.25	2.82	6.92E-06	0.50	0.99	-0.33	0.49	4.87E-01	-2.42	1.88	2.34E-01
rs7094630	10	23,471,878	SBP	0.13	0.88	0.69	1.07	5.14E-01	17.02	5.07	1.69E-02	0.45	0.99	1.33	0.93	3.03E-01	-3.23	3.75	4.77E-01
rs7094630	10	23,471,878	DBP	0.13	0.88	-0.39	0.60	5.13E-01	13.25	2.82	6.93E-06	0.45	0.99	0.49	0.52	3.28E-01	2.64	2.10	2.43E-01
rs2057603	10	23,472,245	SBP	0.13	0.91	0.69	1.07	5.14E-01	17.02	5.07	1.69E-02	0.39	0.99	1.70	0.93	1.49E-01	-6.30	3.77	1.87E-01
rs2057603	10	23,472,245	DBP	0.13	0.91	-0.39	0.60	5.14E-01	13.25	2.82	6.95E-06	0.39	0.99	0.36	0.51	4.69E-01	2.64	2.07	2.39E-01
rs2209501	10	23,489,620	SBP	0.14	NA	0.74	1.09	4.98E-01	17.08	5.21	1.96E-02	0.38	0.93	-0.55	0.90	6.21E-01	0.79	3.50	9.15E-01
rs2209501	10	23,489,620	DBP	0.14	NA	-0.44	0.61	4.74E-01	13.52	2.90	7.91E-06	0.50	0.93	-0.14	0.50	7.65E-01	-2.20	1.92	2.90E-01
rs4492852	11	50,499,436	SBP	0.40	0.95	0.48	0.76	5.26E-01	1.01	3.99	8.57E-01	0.49	0.99	0.86	0.91	4.57E-01	-15.45	2.88	3.93E-06
rs4492852	11	50,499,436	DBP	0.40	0.95	0.37	0.43	3.82E-01	0.61	2.22	7.92E-01	0.49	0.99	0.10	0.51	8.36E-01	-5.23	1.60	2.53E-03
rs8186106	11	50,508,530	SBP	0.30	0.94	-0.56	0.82	4.88E-01	-6.37	4.35	2.98E-01	0.47	0.94	-0.75	0.92	5.28E-01	15.09	2.92	8.92E-06
rs8186106	11	50,508,530	DBP	0.30	0.94	-0.17	0.46	7.10E-01	-2.22	2.42	3.81E-01	0.47	0.94	0.09	0.51	8.62E-01	5.00	1.63	4.59E-03

The role of SNP-loop diuretic interactions in hypertension across ethnic groups in HyperGEN

Supplement Table 4. Cross-Race Comparisons of 100 Top-Ranked SNPs with Suggestive Association for SBP and DBP in African Americans and European Americans; Interaction Effect in the Presence of the Main Effect.

RS Number	Chrom	Physical Postion	Trait	African American								European American							
				MAF	r^2	SNP Main			SNP-Loop Interaction Effect			MAF	r^2	SNP Main			SNP-Loop Interaction Effect		
						Beta	SE	Adjusted *P*-Value	Beta	SE	Adjusted *P*-Value			Beta	SE	Adjusted *P*-Value	Beta	SE	Adjusted *P*-Value
rs4417255	11	51,061,717	SBP	0.26	NA	-0.38	0.82	6.39E-01	-5.86	4.25	3.27E-01	0.44	NA	-0.62	0.90	5.12E-01	15.40	2.92	4.47E-06
rs4417255	11	51,061,717	DBP	0.26	NA	-0.05	0.46	9.19E-01	-1.33	2.37	5.90E-01	0.44	NA	0.01	0.50	9.87E-01	4.85	1.62	5.65E-03
rs7294157	11	51,066,298	SBP	0.26	0.93	-0.44	0.84	5.93E-01	-5.77	4.28	3.38E-01	0.46	0.99	-0.38	0.93	7.28E-01	15.22	3.00	9.77E-06
rs7294157	11	51,066,298	DBP	0.26	0.93	-0.17	0.47	7.17E-01	-1.17	2.38	6.37E-01	0.46	0.99	0.08	0.51	8.65E-01	5.17	1.69	4.63E-03
rs4323853	11	51,077,585	SBP	0.26	0.93	-0.44	0.84	5.94E-01	-5.77	4.28	3.38E-01	0.46	0.99	-0.38	0.93	7.28E-01	15.22	3.00	9.73E-06
rs4323853	11	51,077,585	DBP	0.26	0.93	-0.17	0.47	7.18E-01	-1.18	2.38	6.37E-01	0.46	0.99	0.09	0.51	8.65E-01	5.18	1.69	4.63E-03
rs4480530	11	51,089,764	SBP	0.26	0.93	-0.44	0.84	5.95E-01	-5.78	4.28	3.37E-01	0.46	0.99	-0.38	0.93	7.28E-01	15.23	3.00	9.54E-06
rs4480530	11	51,089,764	DBP	0.26	0.93	-0.17	0.47	7.21E-01	-1.18	2.38	6.35E-01	0.46	0.99	0.09	0.51	8.63E-01	5.18	1.69	4.58E-03
rs12287466	11	51,096,187	SBP	0.26	0.93	-0.44	0.84	5.98E-01	-5.79	4.28	3.35E-01	0.46	0.99	-0.38	0.93	7.28E-01	15.24	3.00	9.41E-06
rs12287466	11	51,096,187	DBP	0.26	0.93	-0.16	0.47	7.25E-01	-1.19	2.38	6.32E-01	0.46	0.99	0.09	0.51	8.63E-01	5.18	1.69	4.56E-03
rs2186641	11	51,163,494	SBP	0.26	0.91	-0.43	0.84	6.08E-01	-5.82	4.27	3.32E-01	0.46	0.99	-0.34	0.93	7.70E-01	15.98	3.08	6.68E-06
rs2186641	11	51,163,494	DBP	0.26	0.91	-0.15	0.47	7.40E-01	-1.22	2.37	6.23E-01	0.46	0.99	0.37	0.52	4.63E-01	5.18	1.73	5.46E-03
rs1694607	11	51,231,886	SBP	0.27	NA	-0.28	0.82	7.29E-01	-5.95	4.25	3.20E-01	0.45	NA	0.59	0.90	5.46E-01	-15.03	2.95	9.61E-06
rs1694607	11	51,231,886	DBP	0.27	NA	0.07	0.46	8.83E-01	-1.43	2.37	5.63E-01	0.45	NA	-0.07	0.50	8.87E-01	-4.64	1.64	8.90E-03
rs1791422	11	51,235,092	SBP	0.27	0.96	-0.37	0.82	6.51E-01	-5.89	4.26	3.25E-01	0.47	0.99	-0.50	0.90	6.59E-01	15.65	2.87	2.19E-06
rs1791422	11	51,235,092	DBP	0.27	0.96	0.02	0.46	9.72E-01	-1.38	2.37	5.77E-01	0.47	0.99	0.10	0.50	8.41E-01	5.35	1.61	2.06E-03
rs554731	11	51,244,515	SBP	0.27	0.96	-0.37	0.82	6.52E-01	-5.89	4.25	3.25E-01	0.47	0.99	-0.49	0.90	6.61E-01	15.65	2.87	2.14E-06
rs554731	11	51,244,515	DBP	0.27	0.96	0.02	0.46	9.70E-01	-1.38	2.37	5.76E-01	0.47	0.99	0.10	0.50	8.41E-01	5.35	1.61	2.04E-03
rs572049	11	51,250,799	SBP	0.27	0.95	-0.37	0.82	6.51E-01	-5.89	4.25	3.24E-01	0.47	0.99	-0.50	0.91	6.60E-01	15.59	2.88	2.58E-06
rs572049	11	51,250,799	DBP	0.27	0.95	0.02	0.46	9.69E-01	-1.39	2.37	5.75E-01	0.47	0.99	0.11	0.50	8.25E-01	5.30	1.61	2.31E-03
rs1791428	11	51,256,936	SBP	0.27	0.96	-0.37	0.82	6.51E-01	-5.89	4.25	3.24E-01	0.47	0.99	-0.52	0.90	6.44E-01	15.57	2.87	2.49E-06
rs1791428	11	51,256,936	DBP	0.27	0.96	0.02	0.46	9.69E-01	-1.39	2.37	5.75E-01	0.47	0.99	0.08	0.50	8.71E-01	5.32	1.61	2.15E-03
rs1391579	11	51,272,254	SBP	0.27	0.97	-0.37	0.82	6.50E-01	-5.89	4.25	3.24E-01	0.47	0.99	-0.54	0.90	6.25E-01	15.37	2.87	3.55E-06
rs1391579	11	51,272,254	DBP	0.27	0.97	0.02	0.46	9.69E-01	-1.39	2.37	5.75E-01	0.47	0.99	0.06	0.50	9.03E-01	5.26	1.61	2.43E-03
rs540337	11	51,306,657	SBP	0.27	0.97	-0.38	0.82	6.43E-01	-5.87	4.25	3.26E-01	0.47	0.99	-0.60	0.90	5.79E-01	14.90	2.87	7.39E-06
rs540337	11	51,306,657	DBP	0.27	0.97	0.02	0.46	9.70E-01	-1.39	2.37	5.75E-01	0.47	0.99	0.01	0.50	9.78E-01	5.12	1.61	3.19E-03
rs1791436	11	51,326,365	SBP	0.27	0.97	-0.39	0.82	6.36E-01	-5.87	4.26	3.27E-01	0.47	0.99	-0.65	0.91	5.52E-01	14.90	2.87	7.47E-06
rs1791436	11	51,326,365	DBP	0.27	0.97	0.01	0.46	9.79E-01	-1.38	2.37	5.76E-01	0.47	0.99	0.04	0.50	9.37E-01	5.07	1.61	3.45E-03
rs7926161	11	51,337,266	SBP	0.27	0.97	-0.44	0.83	5.88E-01	-5.82	4.26	3.31E-01	0.47	0.99	-0.64	0.91	5.54E-01	15.06	2.88	7.30E-06
rs7926161	11	51,337,266	DBP	0.27	0.97	0.00	0.46	9.92E-01	-1.37	2.37	5.81E-01	0.47	0.99	0.04	0.50	9.34E-01	5.05	1.61	3.57E-03
rs4085787	11	51,343,547	SBP	0.27	0.97	-0.45	0.83	5.87E-01	-5.82	4.26	3.31E-01	0.47	0.99	-0.63	0.91	5.56E-01	15.06	2.88	7.36E-06
rs4085787	11	51,343,547	DBP	0.27	0.97	-0.01	0.46	9.90E-01	-1.37	2.37	5.81E-01	0.47	0.99	0.04	0.50	9.31E-01	5.05	1.61	3.59E-03
rs10794396	11	51,371,558	SBP	0.27	0.96	-0.40	0.83	6.30E-01	-5.86	4.26	3.28E-01	0.47	0.98	-0.63	0.91	5.67E-01	14.90	2.88	7.71E-06
rs10794396	11	51,371,558	DBP	0.27	0.96	0.00	0.46	9.98E-01	-1.37	2.37	5.80E-01	0.47	0.98	0.05	0.50	9.22E-01	5.07	1.61	3.56E-03
rs2078611	11	51,383,331	SBP	0.27	0.96	-0.40	0.83	6.28E-01	-5.87	4.27	3.28E-01	0.47	0.98	-0.63	0.91	5.73E-01	14.95	2.89	7.56E-06

The role of SNP-loop diuretic interactions in hypertension across ethnic groups in HyperGEN

Supplement Table 4. Cross-Race Comparisons of 100 Top-Ranked SNPs with Suggestive Association for SBP and DBP in African Americans and European Americans; Interaction Effect in the Presence of the Main Effect.

RS Number	Chrom	Physical Postion	Trait	African American								European American							
				MAF	r^2	SNP Main			SNP-Loop Interaction Effect			MAF	r^2	SNP Main			SNP-Loop Interaction Effect		
						Beta	SE	Adjusted *P*-Value	Beta	SE	Adjusted *P*-Value			Beta	SE	Adjusted *P*-Value	Beta	SE	Adjusted *P*-Value
rs2078611	11	51,383,331	DBP	0.27	0.96	-0.01	0.47	9.89E-01	-1.37	2.37	5.81E-01	0.47	0.98	0.04	0.50	9.36E-01	5.09	1.62	3.53E-03
rs10794400	11	51,402,756	SBP	0.27	0.96	-0.40	0.84	6.27E-01	-5.87	4.27	3.28E-01	0.47	0.98	-0.62	0.91	5.80E-01	14.99	2.89	7.44E-06
rs10794400	11	51,402,756	DBP	0.27	0.96	-0.01	0.47	9.83E-01	-1.37	2.37	5.81E-01	0.47	0.98	0.03	0.50	9.46E-01	5.10	1.62	3.51E-03
rs3741378	11	65,165,513	SBP	0.14	0.83	1.24	1.12	2.65E-01	1.96	5.86	8.12E-01	0.29	0.90	-1.67	1.06	1.53E-01	-17.37	3.55	9.73E-06
rs3741378	11	65,165,513	DBP	0.14	0.83	-0.07	0.63	9.13E-01	0.92	3.26	7.87E-01	0.29	0.90	-0.86	0.59	1.33E-01	-6.64	1.98	1.86E-03
rs17331385	11	103,431,386	SBP	0.23	0.90	0.65	0.94	4.87E-01	-10.30	4.14	7.69E-02	0.12	0.87	4.47	1.45	4.27E-03	-18.69	4.66	6.29E-04
rs17331385	11	103,431,386	DBP	0.23	0.90	0.71	0.52	1.78E-01	-7.16	2.31	2.97E-03	0.12	0.87	2.66	0.80	6.29E-04	-12.09	2.52	8.76E-06
rs10895567	11	103,436,314	SBP	0.28	0.95	-0.21	0.81	7.95E-01	-8.22	3.68	1.12E-01	0.14	0.99	3.94	1.34	4.25E-03	-18.15	4.45	4.39E-04
rs10895567	11	103,436,314	DBP	0.28	0.95	0.09	0.45	8.46E-01	-4.95	2.05	2.08E-02	0.14	0.99	2.35	0.74	1.07E-03	-11.56	2.41	8.72E-06
rs17655864	11	110,336,581	SBP	0.16	0.79	-0.45	1.03	6.60E-01	-8.78	4.75	1.89E-01	0.06	0.97	3.09	2.16	2.30E-01	-31.30	7.64	1.44E-04
rs17655864	11	110,336,581	DBP	0.16	0.79	-1.50	0.57	9.07E-03	-1.21	2.65	6.62E-01	0.06	0.97	3.12	1.20	7.67E-03	-22.10	4.34	2.31E-06
rs1946518	11	111,540,668	SBP	0.41	0.96	-0.74	0.75	3.21E-01	-0.14	3.10	9.73E-01	0.35	0.98	0.39	0.95	6.89E-01	-13.02	3.19	1.86E-04
rs1946518	11	111,540,668	DBP	0.41	0.96	-0.57	0.42	1.70E-01	0.53	1.72	7.68E-01	0.35	0.98	0.63	0.52	2.16E-01	-9.20	1.82	2.84E-06
rs1946519	11	111,540,717	SBP	0.41	0.95	-0.69	0.75	3.55E-01	-0.11	3.12	9.80E-01	0.35	0.97	0.36	0.95	7.12E-01	-12.97	3.20	2.03E-04
rs1946519	11	111,540,717	DBP	0.41	0.95	-0.51	0.42	2.23E-01	0.54	1.74	7.65E-01	0.35	0.97	0.62	0.53	2.24E-01	-9.19	1.83	3.16E-06
rs2728821	12	22,353,878	SBP	0.46	0.81	0.06	0.92	9.46E-01	7.65	3.67	1.38E-01	0.26	0.62	0.06	1.11	9.64E-01	-21.98	4.33	7.86E-06
rs2728821	12	22,353,878	DBP	0.46	0.81	-0.52	0.52	3.16E-01	4.60	2.04	3.12E-02	0.26	0.62	0.24	0.61	6.87E-01	-7.63	2.39	3.07E-03
rs1568031	12	122,702,560	SBP	0.02	0.97	1.48	2.92	6.10E-01	-1.44	7.60	8.92E-01	0.16	1.00	-0.11	1.25	8.44E-01	-24.29	4.88	9.39E-06
rs1568031	12	122,702,560	DBP	0.02	0.97	-0.83	1.63	6.12E-01	2.88	4.22	5.14E-01	0.16	1.00	0.20	0.69	7.62E-01	-7.99	2.71	6.27E-03
rs11573005	12	122,710,232	SBP	0.02	0.97	1.47	2.92	6.13E-01	-1.43	7.60	8.93E-01	0.16	1.00	-0.19	1.25	8.86E-01	-24.08	4.82	8.69E-06
rs11573005	12	122,710,232	DBP	0.02	0.97	-0.83	1.63	6.10E-01	2.88	4.22	5.13E-01	0.16	1.00	0.19	0.69	7.78E-01	-8.23	2.68	4.36E-03
rs3890823	12	130,301,745	SBP	0.03	0.92	0.17	2.25	9.40E-01	-54.40	12.42	1.83E-03	0.27	0.97	0.57	1.07	8.19E-01	2.93	3.38	4.34E-01
rs3890823	12	130,301,745	DBP	0.03	0.92	2.75	1.26	2.87E-02	-33.28	6.92	4.13E-06	0.27	0.97	-0.15	0.59	7.90E-01	4.70	1.84	1.79E-02
rs12303986	12	130,314,492	SBP	0.03	NA	-0.68	2.15	7.49E-01	54.17	12.25	1.65E-03	0.43	NA	0.36	0.92	8.54E-01	-0.53	2.94	8.27E-01
rs12303986	12	130,314,492	DBP	0.03	NA	-3.04	1.20	1.11E-02	33.16	6.82	3.28E-06	0.50	NA	-0.12	0.51	8.11E-01	1.35	1.61	4.37E-01
rs9568642	13	51,090,690	SBP	0.47	0.82	-1.03	0.78	1.86E-01	10.43	3.42	3.02E-02	0.13	0.91	-1.15	1.48	5.78E-01	22.47	4.81	3.17E-06
rs9568642	13	51,090,690	DBP	0.47	0.82	0.20	0.44	6.45E-01	1.34	1.90	4.99E-01	0.13	0.91	-0.30	0.81	7.02E-01	10.19	2.83	8.35E-04
rs927518	13	52,688,634	SBP	0.44	0.96	0.99	0.73	1.69E-01	2.39	3.20	5.95E-01	0.38	0.99	0.20	0.96	8.87E-01	15.07	3.04	8.97E-06
rs927518	13	52,688,634	DBP	0.44	0.96	-0.01	0.40	9.83E-01	1.67	1.78	3.70E-01	0.38	0.99	-0.38	0.53	4.61E-01	6.71	1.69	2.39E-04
rs12430231	13	64,347,888	SBP	0.13	0.86	2.96	1.12	7.79E-03	-3.09	4.66	6.37E-01	0.14	0.93	3.50	1.39	1.98E-02	-27.01	4.92	2.12E-06
rs12430231	13	64,347,888	DBP	0.13	0.86	0.83	0.62	1.82E-01	-0.11	2.59	9.66E-01	0.14	0.93	1.71	0.77	2.21E-02	-6.77	2.72	2.10E-02
rs9558048	13	102,753,876	SBP	0.19	0.81	0.41	0.94	6.63E-01	3.80	4.04	5.04E-01	0.23	0.99	1.28	1.16	3.78E-01	-20.39	4.05	1.25E-05
rs9558048	13	102,753,876	DBP	0.19	0.81	-0.63	0.52	2.33E-01	1.45	2.25	5.37E-01	0.23	0.99	1.39	0.64	2.66E-02	-10.73	2.24	9.28E-06
rs2570124	15	85,439,847	SBP	0.32	0.96	0.17	0.81	8.35E-01	-8.36	4.32	1.69E-01	0.21	0.89	-1.71	1.09	1.86E-01	21.12	4.07	7.10E-06
rs2570124	15	85,439,847	DBP	0.32	0.96	-0.07	0.45	8.86E-01	-3.67	2.40	1.44E-01	0.21	0.89	0.09	0.60	8.76E-01	4.83	2.23	4.50E-02

Supplement Table 4. Cross-Race Comparisons of 100 Top-Ranked SNPs with Suggestive Association for SBP and DBP in African Americans and European Americans; Interaction Effect in the Presence of the Main Effect.

RS Number	Chrom	Physical Postion	Trait	African American								European American							
				MAF	r^2	SNP Main			SNP-Loop Interaction Effect			MAF	r^2	SNP Main			SNP-Loop Interaction Effect		
						Beta	SE	Adjusted *P* -Value	Beta	SE	Adjusted *P* -Value			Beta	SE	Adjusted *P* -Value	Beta	SE	Adjusted *P* -Value
rs1429935	18	19,340,214	SBP	0.47	0.95	1.54	0.75	3.90E-02	-8.58	3.44	7.62E-02	0.48	0.96	2.78	0.92	3.39E-03	-15.70	3.20	7.63E-06
rs1429935	18	19,340,214	DBP	0.47	0.95	0.85	0.42	4.26E-02	-2.33	1.91	2.44E-01	0.48	0.96	1.87	0.50	1.41E-04	-6.98	1.76	2.47E-04
rs6507708	18	19,355,454	SBP	0.48	0.96	1.52	0.75	4.28E-02	-8.60	3.44	7.51E-02	0.49	0.95	2.65	0.91	5.08E-03	-15.95	3.19	5.57E-06
rs6507708	18	19,355,454	DBP	0.48	0.96	0.77	0.42	6.65E-02	-2.23	1.91	2.65E-01	0.49	0.95	1.86	0.50	1.43E-04	-7.18	1.76	1.53E-04
rs12964689	18	19,370,996	SBP	0.46	0.97	1.71	0.76	2.27E-02	-9.44	3.60	6.19E-02	0.49	0.95	2.80	0.90	3.29E-03	-16.70	3.17	1.86E-06
rs12964689	18	19,370,996	DBP	0.46	0.97	0.94	0.42	2.63E-02	-1.92	2.00	3.60E-01	0.49	0.95	1.93	0.50	6.85E-05	-7.26	1.75	1.17E-04
rs504368	18	53,933,770	SBP	0.44	0.39	1.50	1.15	1.88E-01	5.62	5.25	4.47E-01	0.17	0.42	1.20	1.89	6.10E-01	-30.50	5.85	6.89E-06
rs504368	18	53,933,770	DBP	0.44	0.39	0.32	0.64	6.21E-01	2.58	2.92	3.97E-01	0.17	0.42	-0.63	1.04	5.33E-01	-7.65	3.19	2.61E-02
rs8094402	18	72,836,437	SBP	0.34	0.75	0.11	0.86	9.01E-01	0.04	4.18	9.95E-01	0.10	0.80	-1.99	1.71	3.64E-01	-16.38	6.00	1.11E-02
rs8094402	18	72,836,437	DBP	0.34	0.75	0.27	0.48	5.79E-01	-1.40	2.32	5.66E-01	0.10	0.80	-0.35	0.94	7.00E-01	-16.01	3.30	6.72E-06
rs6012061	20	44,940,236	SBP	0.04	0.84	3.42	2.33	1.40E-01	-46.16	10.17	1.25E-03	0.11	0.69	3.20	1.53	5.00E-02	-10.88	5.02	6.61E-02
rs6012061	20	44,940,236	DBP	0.04	0.84	2.06	1.30	1.12E-01	-27.26	5.66	4.06E-06	0.11	0.69	1.68	0.84	3.92E-02	-4.68	2.75	1.15E-01
rs7274151	20	44,962,232	SBP	0.04	0.73	3.47	2.09	9.44E-02	-41.98	9.53	1.73E-03	0.08	0.75	-0.12	1.89	9.57E-01	-3.46	6.14	5.63E-01
rs7274151	20	44,962,232	DBP	0.04	0.73	1.77	1.16	1.30E-01	-24.65	5.30	8.70E-06	0.08	0.75	-1.16	1.04	2.53E-01	-2.50	3.29	4.81E-01
rs7261412	20	44,963,118	SBP	0.04	0.81	3.46	2.08	9.38E-02	-41.82	9.51	1.76E-03	0.12	0.76	3.21	1.52	4.93E-02	-10.47	5.06	7.88E-02
rs7261412	20	44,963,118	DBP	0.04	0.81	1.76	1.16	1.29E-01	-24.56	5.29	9.01E-06	0.12	0.76	1.51	0.84	6.28E-02	-4.49	2.77	1.33E-01
rs6094522	20	45,005,227	SBP	0.09	0.79	2.54	1.30	4.84E-02	-35.86	8.07	1.56E-03	0.17	0.95	-0.76	1.32	6.52E-01	0.35	4.54	9.63E-01
rs6094522	20	45,005,227	DBP	0.09	0.79	1.44	0.72	4.62E-02	-20.74	4.49	9.80E-06	0.17	0.95	-0.30	0.72	6.67E-01	-0.09	2.48	9.73E-01
